# User Experience and Early Clinical Outcomes of a Mental Wellness Chatbot for Depression and Anxiety: Pilot Evaluation Mixed Methods Study

**DOI:** 10.2196/90644

**Published:** 2026-04-14

**Authors:** Scott Graupensperger, Emily J Ward, Graham Baum, Kate H Bentley, Emily R Dworkin, Millard Brown, Adam Chekroud, Matt Hawrilenko

**Affiliations:** 1 Spring Health, 60 Madison Ave, New York, NY, 10010, United States, 1 855-629-0554; 2 Psychiatry, School of Medicine, Yale University, New Haven, CT, United States

**Keywords:** digital health, artificial intelligence, large language models, therapeutic alliance, mental health.

## Abstract

**Background:**

Artificial intelligence–powered conversational agents (ie, chatbots) are increasingly popular outlets for users seeking psychological support, yet little is known about how users experience early-stage prototypes or which therapeutic processes contribute to clinical improvement. A transparent evaluation of emerging chatbot prototypes is needed to clarify if, how, and why artificial intelligence companions work and to guide their continued development.

**Objective:**

This mixed methods pilot study evaluated user experience, acceptability, and preliminary clinical signals for an early-stage mental wellness chatbot. We also examined whether baseline symptom severity moderated clinical improvement.

**Methods:**

Three sequential cohorts (n=125) completed a 2-week, incentivized chatbot exposure (approximately 60 min per week). Participants provided first-impression ratings, qualitative feedback, and pre–post assessments of depressive symptoms (PHQ-8 [Patient Health Questionnaire-8]), anxiety symptoms (GAD-7 [Generalized Anxiety Disorder-7]), psychological distress, well-being, and loneliness. Statistical models estimated symptom change and tested interactions with baseline symptom severity. Mixed methods analysis integrated quantitative outcomes with large language model–assisted qualitative content analysis of open-ended responses.

**Results:**

Participants described the chatbot as accessible, easy to use, and emotionally validating, while citing limitations in personalization and conversational depth. Qualitative responses consistently highlighted early therapeutic processes such as emotional validation, goal setting, and perceived attunement. Regression models showed significant pre–post reductions in depressive (Hedges *g*=–0.32) and anxiety (*g*=–0.32) symptoms, alongside modest improvements in distress and well-being. Baseline severity moderated improvement, with marginal effects indicating larger predicted reductions at higher PHQ-8 and GAD-7 baseline scores (eg, PHQ-8=15: *g*=–0.84; GAD-7=15: *g*=–0.62).

**Conclusions:**

This pilot provides a comprehensive view of early chatbot development and suggests promising user experiences and preliminary symptom improvements under structured pilot conditions. By integrating experiential and exploratory clinical data, the study identifies candidate process targets to inform ongoing refinement. Findings support continued development and demonstrate procedural feasibility for progression to larger, longer-term trials evaluating engagement and clinical outcomes under more naturalistic conditions.

## Introduction

### Background

Mental health conditions such as depression and anxiety remain among the leading causes of disability worldwide, yet access to timely, evidence-based care continues to fall short of population need [1]. Structural barriers such as high costs, shortages of trained clinicians, long waitlists, geographic disparities, and perceived stigma limit the reach of traditional psychotherapy and contribute to persistent treatment gaps [2,3]. As a result, scalable digital solutions have become a major focus for innovation, with particular attention to platforms that can deliver immediate, flexible, and low-threshold support outside of, or as a supplement to, formal clinical settings.

Large language model (LLM)–based chatbots are rapidly evolving in the digital mental health space [4]. As reviewed in recent narrative and systematic syntheses of artificial intelligence (AI) mental health chatbots [5,6], these chatbots offer the possibility of responsive, personalized conversational support delivered at scale, with emerging studies suggesting they may help reduce mental health symptoms, increase engagement, and broaden access to care [7-13]. Yet, some researchers have been critical of diving straight into clinical trials [14], as the development of these products has largely outpaced the empirical evidence base, with key questions remaining about how therapeutic processes unfold within chatbot interactions, how users perceive safety and trust, and whether these tools function best as stand-alone interventions or complements to clinician-delivered care [15,16].

To date, studies assessing chatbots as mental health tools provide limited transparency into how prototypes were designed, refined, and evaluated for safety. As a result, little is known about how users actually experience early-stage chatbots, such as how safe, helpful, or trustworthy they feel, what facilitates or impedes engagement, and how these factors affect the impact of the chatbot. This gap is consequential, as understanding users’ initial experiences with mental wellness chatbot prototypes is essential for responsible development and real-world effectiveness. Indeed, prior research on digital mental health interventions shows that early impressions such as perceived safety, personalization, and feeling understood are strongly associated with subsequent engagement, adherence, and indicators of therapeutic response [17,18]. These experiential factors also shape trust and therapeutic alliance, which are central determinants of whether users continue to engage long enough to experience meaningful benefits [19,20]. However, recent theoretical work suggests that alliance and other change processes in chatbot-based interventions may not operate identically to those in human psychotherapy, given that chatbots lack human therapists’ ontological and sociocultural status [21]. This further highlights the need to empirically examine how users experience and interpret interactions with early-stage mental wellness chatbots.

Whereas several recent studies have focused on symptom outcomes in randomized trials [5] or evaluated chatbot performance in controlled vignette-based simulations [22], fewer investigations have examined early-stage prototype development using mixed methods approaches that integrate user experience, safety workflows, and preliminary clinical signals. As a result, there remains limited visibility into how conversational design choices, early user impressions, and structured protocols shape engagement and short-term outcomes. Because complex, multidimensional user experiences are best captured using human-centered, mixed methods that combine quantitative usability and symptom metrics with qualitative insight into how users interpret and experience emerging AI tools [23], early-stage evaluations benefit from integrating both forms of evidence. This study provides a comprehensive assessment of an early-stage chatbot prototype, explicitly linking user experience and exploratory clinical outcomes to inform responsible refinement and progression to larger trials.

### This Study

Situated within digital mental health evaluation research, this pilot study used mixed methods to evaluate an early-stage LLM–based mental wellness chatbot designed to support adults experiencing at least mild anxiety or depression. Across 3 successive cohorts, participants interacted with the chatbot over a 2-week period, providing both quantitative and qualitative feedback on usability, acceptability, and perceived therapeutic value. We also explored pre-post changes in mental health symptoms as a preliminary gauge for a potential therapeutic signal. In the absence of randomization or a comparison arm, early clinical signals from chatbot prototypes should be interpreted as exploratory rather than as evidence of efficacy [24]. By integrating experiential and early clinical outcome data within a structured pilot framework, this study contributes prototype-stage evidence to inform responsible refinement, optimization, and future controlled evaluation of mental wellness chatbots. Accordingly, the aim of this study was to conduct a comprehensive mixed methods evaluation of a generative LLM–based mental wellness chatbot, examining user experience, safety monitoring feasibility, and exploratory clinical signal to inform responsible prototype refinement and future controlled trials.

## Methods

### Overview of the AI Chatbot

The LLM–powered mental wellness chatbot evaluated in this study was developed to provide brief emotional support for adults experiencing mild to moderate anxiety or depressive symptoms. Responses were guided by evidence-based principles drawn from supportive and cognitive-behavioral therapy frameworks, enabling the chatbot to respond empathically to user input while encouraging adaptive coping and reflection.

Rather than delivering a structured, manualized treatment protocol, the chatbot facilitates supportive mental wellness conversations informed by evidence-based common factors, including emotional validation and reflective listening, collaborative clarification of goals, and brief coping or activation prompts (eg, grounding or perspective-taking exercises) [25]. These processes were intentionally scaffolded through system-level instructions emphasizing emotional safety, nondirectiveness, and avoidance of clinical diagnosis or prescriptive advice. The app functioned as a stand-alone iOS application with a cloud-based backend hosted by Amazon Web Services. Additional details regarding the therapeutic framework, AI architecture, and real-time safety classifier are provided in the Multimedia Appendix 1 [26-37].

### Study Procedures

The study was conducted from September to November 2025. Participants were recruited through dscout (dscout Inc), a web-based research platform with a large opt-in panel of adults across the United States that is focused on user experience research. Eligible participants were aged ≥21 years who reported at least mild symptoms of anxiety or depression at the time of screening. Individuals who endorsed suicidal or self-harm thoughts in the past 2 weeks (ie, score of ≥1 on the ninth item of the Patient Health Questionnaire-9 [PHQ-9]) or serious mental illness, as well as those currently receiving psychotherapy, were excluded from this early-stage pilot test of an app that has not yet been evaluated for individuals with complex clinical needs. Additional screening and demographic balance procedures are described in the Multimedia Appendix 1 [26-37]. Enrollment occurred in 3 successive cohorts to allow iterative refinement of the chatbot prototype between cohorts. After completing eligibility screening, participants were invited to complete a baseline survey assessing demographics, clinical symptomatology (eg, depressive and anxiety symptoms), and attitudes toward mental health therapy and chatbots for emotional support. After baseline, participants were instructed to download the prototype application via TestFlight—Apple’s official beta-testing platform for iOS. Participants were instructed to use the chatbot for at least 60 minutes per week during the 2-week study period. The first use of the chatbot triggered a first impressions survey and open-ended qualitative prompts focused on initial user experience feedback. One week following the app download, participants completed a brief follow-up survey and qualitative prompts focused on usability and acceptability. Finally, the 2-week follow-up survey reassessed the clinical metrics and attitudes toward mental wellness chatbots originally assessed at baseline. All survey instruments/measures and interview materials are described in the Multimedia Appendix 1 [26-37].

Chatbot interactions were continuously monitored in real-time by an automated, transcript-based safety classifier that was calibrated to high sensitivity due to the early stage of chatbot testing. The classifier detects participant responses indicating potential harm to self, to others, or from others (Multimedia Appendix 1 [26-37] provides details). All flagged responses triggered a real-time alert to the study clinicians, who reviewed each flagged transcript within 2 hours to determine whether phone-based outreach for a safety check or emergency intervention was warranted.

### Participants

The screening survey was hosted on dscout and distributed to their active panel of participants. As required by dscout, the study description made clear that this was a 2-week digital mental health and well-being study involving the use of a mobile app. The screener stopped participants as soon as they failed an eligibility criterion to reduce the burden of continuing to ask unnecessary items once ineligibility was established. Because exclusions occurred dynamically throughout the screener, precise counts for every individual exclusion step cannot be calculated.

A detailed description of eligibility screening and a participant flow diagram is provided in Figure S2 in Multimedia Appendix 1 [26-37]. In short, 3406 individuals completed the screening, and 184 (5.4%) were eligible, with the most common exclusions being living in an ineligible state, not meeting the minimum symptom severity threshold (PHQ-2 or Generalized Anxiety Disorder-2 [GAD-2] score ≥3), or being currently engaged in some form of mental health care. Of the eligible pool, 125 were ultimately enrolled. Consistent with the aims of pilot-stage testing, this sample was not designed to represent individuals with acute or high-risk clinical presentations. The final sample represented 32 different US states, ranging in age from 21 to 67 (mean 34.6*,* SD 9.9) years, and 80 (64%) identified as women. While most reported being White (74/125, 59.2%), 19 (15.2%) were Black or African American, 13 (10.4%) were Asian or Asian American, 10 (8%) were multiple races, and 9 (7.2%) reported another race or preferred not to answer. Regarding ethnicity, 13 (10.4%) were Hispanic/Latinx. Most were employed (91/125, 72.8%), while 17 (13.6%) listed their employment status as currently a student, and 17 (13.6%) were not employed. More than half (70/125, 56%) reported some form of prior mental health care. While 89 (74.2%) had previously used an AI chatbot, only 30 (25.2%) had used one for mental health support or information.

### Ethical Considerations

This study was approved by Pearl IRB (Indianapolis, IN; Study# 2025‐0463), an independent institutional review board. All procedures were conducted in accordance with the ethical standards of the 1964 Helsinki Declaration and its later amendments. Consent was obtained electronically from all participants prior to participation. Usage was incentivized at US $50 per week for those who met the 60-minute threshold and US $30 for those who exceeded 30 minutes but did not reach 60 minutes. Including compensation for survey and interview completion, participants could earn up to US $275 in total. All quantitative and qualitative data were deidentified prior to analysis and stored on secure, access-restricted servers. The secure LLM (GPT-4o-mini) instance used for qualitative analysis did not retain participant data or use it for model training. Only authorized members of the research team had access to study data.

### Qualitative Analytic Approach

Open-ended written responses and transcribed video responses were analyzed using a structured, LLM-assisted qualitative content analysis approach designed to characterize recurring feedback patterns within qualitative data. LLM-assisted content analysis is an emergent qualitative technique that leverages LLMs’ strengths in pattern recognition and linguistic synthesis [38]. An inductive approach was appropriate given that our goal for this analysis was purely descriptive: that is, we aimed to characterize emergent themes rather than to apply prespecified theories or codes to the data [39]. The LLM was prompted to extract candidate themes related to user experience (both positive and negative) and to provide illustrative quotes directly drawn from participant responses. Themes were generated separately for each cohort to explore potential shifts as the chatbot prototype evolved across iterative refinements.

In keeping with qualitative rigor standards [40], several strategies were used to enhance trustworthiness. A reflexive approach was adopted to account for potential bias arising from the nonneutrality of the LLM and the researchers’ positionality as employees of the company developing the chatbot. To increase dependability, the analytic procedure was repeated independently 3 times using identical prompts, and theme labels and descriptions were compared across runs to assess stability and consistency in structure. To enhance credibility [40], the researchers reviewed LLM-generated themes and quotations to ensure that quotes were coherent, nonredundant, and accurately reflected the described pattern. To ensure confirmability, we retained only themes that were consistently reproduced across model runs and deemed coherent in relation to the dataset as a whole. Transferability was supported by a detailed description of the study context, participant characteristics, and prototype-stage setting, allowing readers to evaluate applicability to similar contexts.

### Statistical Analyses

The goal of this user experience pilot trial was to recruit a sufficiently large sample to characterize usability and acceptability, gather rich user feedback, and obtain preliminary estimates of symptom change to inform the design and justification of future full-scale trials; accordingly, it was not statistically powered to detect clinical effects. First, descriptive statistics were used to summarize responses to the first-impression survey and 1-week follow-up surveys, including items assessing perceived emotional safety, trust, sense of being understood, professionalism, personalization, acceptability, and perceived advantages relative to a human therapist. Mean item ratings and stacked bar distributions were calculated using all available data.

The PHQ-8 and GAD-7 were analyzed separately to capture domain-specific symptom change and also combined into a Patient Health Questionnaire Anxiety and Depression Scale (PHQ-ADS) composite representing overall symptom burden [26], as this is a common metric used in digital mental health studies [41,42]. The composite was included to facilitate interpretation of global symptom change rather than to introduce a distinct confirmatory endpoint. Consistent with the developmental aims of this pilot, all symptom outcomes were interpreted as exploratory indicators to inform future trial design.

Clinical outcome analyses were restricted to participants with at least mild symptoms at baseline for the given outcome. For depressive symptoms, models included only participants with PHQ-8 scores ≥5 at baseline; for anxiety symptoms, only those with baseline GAD-7 scores ≥5 were included. Models estimating total symptoms (PHQ-ADS) and secondary outcomes were estimated among participants who had either PHQ-8 or GAD-7 ≥5 at baseline (not necessarily both). Changes in clinical outcomes from baseline to the 2-week follow-up were estimated using linear mixed-effects models with a random intercept for participant, with time (follow-up vs baseline) as the main predictor and demographic covariates and cohort indicators included as fixed effects. Exploratory regression models tested whether first-impression ratings of the chatbot predicted 1-week therapeutic alliance and acceptability scores, adjusting for covariates and cohort. Finally, Pearson correlations examined associations between each first-impression rating and residualized symptom change scores (ie, calculated by regressing follow-up symptom scores on baseline severity and using the residuals as indices of symptom change independent of baseline levels), providing preliminary evidence for experiential predictors of improvement.

This study was reported in accordance with the CONSORT (Consolidated Standards of Reporting Trials) extension for pilot and feasibility trials (Checklist 1).

## Results

### Study Protocol Feasibility Metrics

#### Overview

The following metrics describe the feasibility of the structured, incentivized study protocol rather than naturalistic engagement patterns. Because app use was incentivized to encourage adequate prototype evaluation, retention, and usage statistics should be interpreted as indicators of procedural feasibility informing next-stage trials, not as estimates of voluntary real-world uptake. Retention was strong under the incentivized, time-limited study protocol (96% at 1 week; 95% at 2 weeks). We nevertheless examined correlates of attrition using logistic regression and found that dropout was unrelated to age, gender, education, employment, household income, baseline symptom levels, or baseline attitudes toward mental wellness chatbots. Across the 3 enrollment cohorts, there were no significant baseline differences in clinical severity.

Over the 2-week intervention period (defined as the first 16 days following activation to accommodate minor variation in start timing), participants completed a median of 7 sessions, with 79.6% completing at least 5 sessions and 30.1% completing at least 10 sessions. Participants used the chatbot on 7.4 distinct days, on average (SD 2.8), indicating that engagement was generally distributed across the exposure period rather than all at once. Across sessions, participants generated a median of 157 talk-turns (ie, message exchanges), indicating sustained conversational engagement with the chatbot. Adherence to the study protocol was high, with 75.2% of participants reaching the recommended exposure (≥120 total minutes of use) and 87.6% completing at least 90 minutes (median 141.9 minutes). Older age was significantly associated with greater total minutes of app use; no other baseline demographic or attitudinal variables were related to minutes of use or total talk turns with the chatbot. Notably, household income was not significantly associated with total minutes of use (Spearman ρ=−0.06, *P*=.57) or with likelihood of reaching the incentivized exposure threshold (odds ratio 1.00, 95% CI 1.00-1.00; *P*=.91).

#### Safety Monitoring

During the 2-week exposure period, 10 (8.0%) participants triggered a real-time safety alert (no participants triggered more than one). Of these 10 alerts, 5 were flagged for potential risk of harm to self, 2 for harm to others, and 3 for harm from others. All transcripts flagged by the real-time safety classifier were reviewed by a study clinician within 2 hours, and none met the study threshold for phone-based safety outreach (eg, indicators of acute or imminent risk). The “harm to self” alerts referenced concerns about death in other contexts (eg, panic attacks, loved ones) or risk factors (eg, feeling like a burden), but no suicidal thoughts. “Harm to others” alerts reflected third-party nonviolent contextual behaviors or hypothetical situations, and “harm from others” alerts reflected general interpersonal conflict or precautionary safety concerns.

### Qualitative User Experience Findings

Consistent with the early-stage, developmental focus of this pilot, qualitative analyses were exploratory and used primarily to identify actionable areas for product refinement. Across the 3 cohorts, participants described the chatbot as accessible, easy to use, and nonjudgmental, but limited by repetitive and impersonal responses. Qualitative content analysis identified three primary domains of experience: (1) accessibility and usability, (2) emotional validation and reflection, and (3) limitations in personalization and conversational depth.

Participants across all cohorts described the chatbot as a convenient outlet for emotional expression and appreciated being able to engage at any time and fit sessions flexibly into their routines. As one participant explained, “The chatbot was able to express my feelings without any judgment, and it’s there whenever I needed to talk.” [Cohort 1]. Others highlighted the app’s intuitive interface and customizable sessions, calling it “super easy to use. To set up... super easy. It may be the simplest app I’ve ever used to actually get in and do that.” [Cohort 2] and appreciating that “it was handy. I could use it at any point in time 24/7.” [Cohort 3]. These qualities made the chatbot particularly appealing as a low-barrier, on-demand support tool.

Across cohorts, participants also valued the chatbot’s ability to validate emotions and encourage self-reflection. Many described feeling heard and supported when the chatbot acknowledged their emotions, with one noting that it “did a good job validating my feelings and helping me remember that others feel the same way” [Cohort 1]. Later users reported that it facilitated goal setting and simple coping strategies, such as reminders to take breaks or practice breathing exercises: “It helped me set a goal, did a grounding exercise, and gave me tools to get started” [Cohort 3]. These elements contributed to perceptions of the chatbot as supportive, though somewhat limited in depth.

Despite these strengths, the chatbot’s repetitiveness and lack of personalization emerged as key pain points. Participants frequently described conversations as “robotic” or “circular,” with limited responsiveness to context. One Cohort 1 user noted, “It just kept asking the same questions and using the same exact language,” while a Cohort 2 participant remarked, “Every response was starting with my name... it seems very unnatural and stiff.” Others lamented that the chatbot failed to pivot or offer specific advice: “It kept asking me the same questions... whereas a human could pivot the conversation” [Cohort 3]. Feedback on session structure also shifted across cohorts. Cohort 1 users requested more guidance around session length, which informed the introduction of timed sessions; however, Cohorts 2 and 3 reported occasional frustration when session cut-offs truncated ongoing discussion.

Across cohorts, core themes were consistent: participants valued accessibility and emotional validation but critiqued repetitive and impersonal responses. Cohort-level differences primarily reflected emphasis on emotional safety in Cohort 1, usability and goal-setting in Cohort 2, and coping tools and session design in Cohort 3. As the prototype evolved across cohorts, feedback appeared to shift from general impressions to more specific critiques of functionality and conversational adaptability. Overall, participants saw clear potential in the chatbot while emphasizing that improvements in personalization, conversational nuance, and responsiveness will be essential for enhancing its value.

### Usability of the Chatbot App

Usability, assessed using an adapted Intervention Usability Scale (IUS) [27] specific to the mental wellness chatbot (0‐100 scale), was generally favorable. Participants reported a mean IUS score of 75.0 (SD 13.6). Although the IUS does not have established percentile norms, scores in the mid-70s are interpreted as “good” usability on the original System Usability Scale [43]. For context, the mean usability rating in this study was higher than that reported for the motivational interviewing intervention in the original IUS validation paper (68.7), suggesting that the chatbot app was, on the whole, experienced as usable by participants. Household income was not correlated with usability scores (*r*=0.07, *P*=.47).

### User Experience and Acceptability Ratings

**Figure 1.**
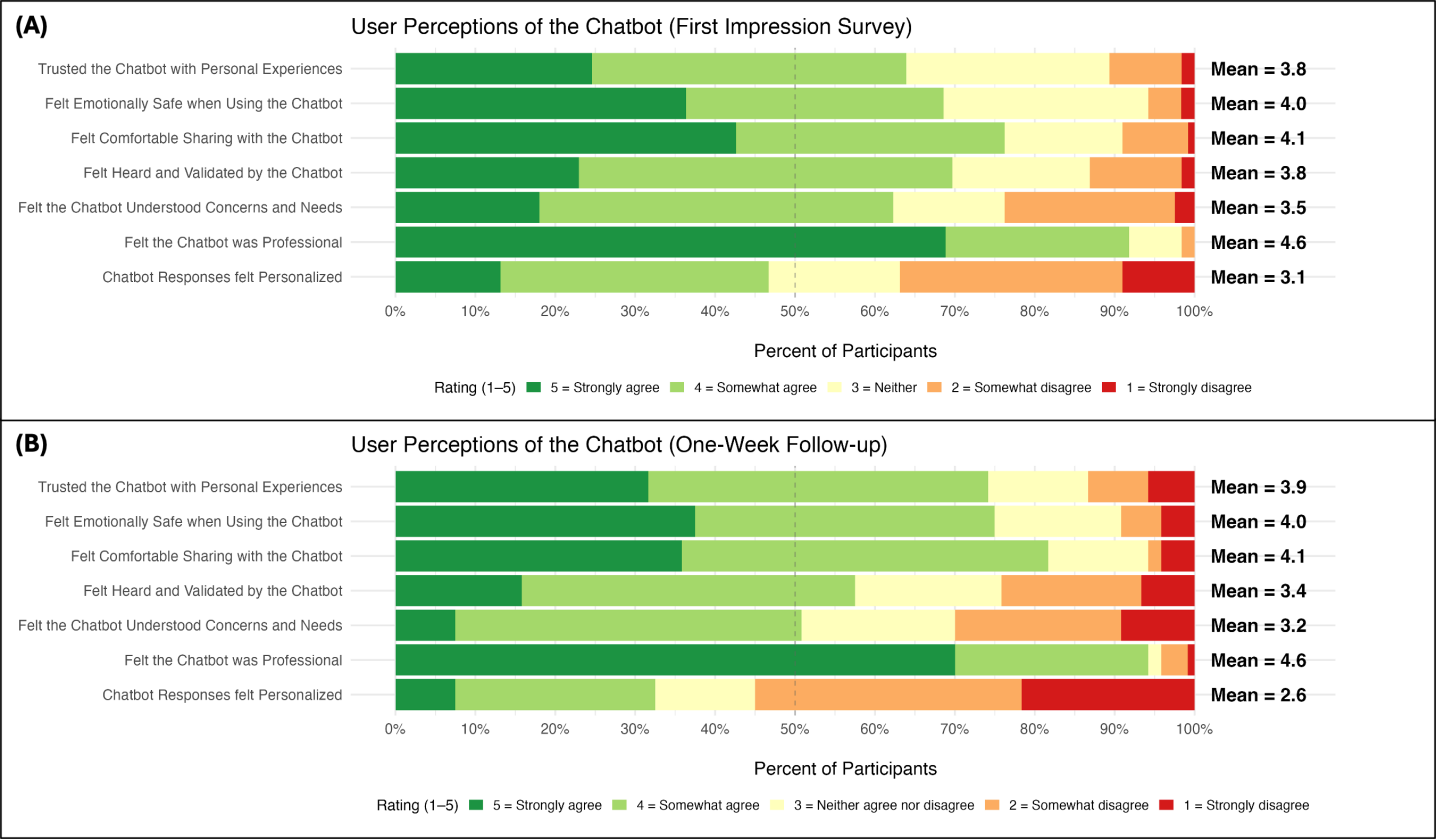
Panel (A) displays ratings from the first-impression survey completed immediately after initial interaction with the chatbot; Panel (B) displays ratings from the 1-week follow-up survey.

Participants generally reported moderately positive first impressions of the chatbot’s emotional safety, trustworthiness, sense of being heard and understood, and professionalism, with mean ratings hovering slightly above the scale midpoint on most items (Figure 1A). However, a substantial minority of participants endorsed neutral or negative response options, particularly for items related to feeling deeply understood (≈40%) and perceived personalization of responses (≈65%), indicating focal opportunities for improvement. At the 1-week follow-up, perceptions of emotional safety, comfort, and professionalism were generally stable (Figure 1B), although ratings of response personalization declined relative to first impressions.

**Figure 2.**
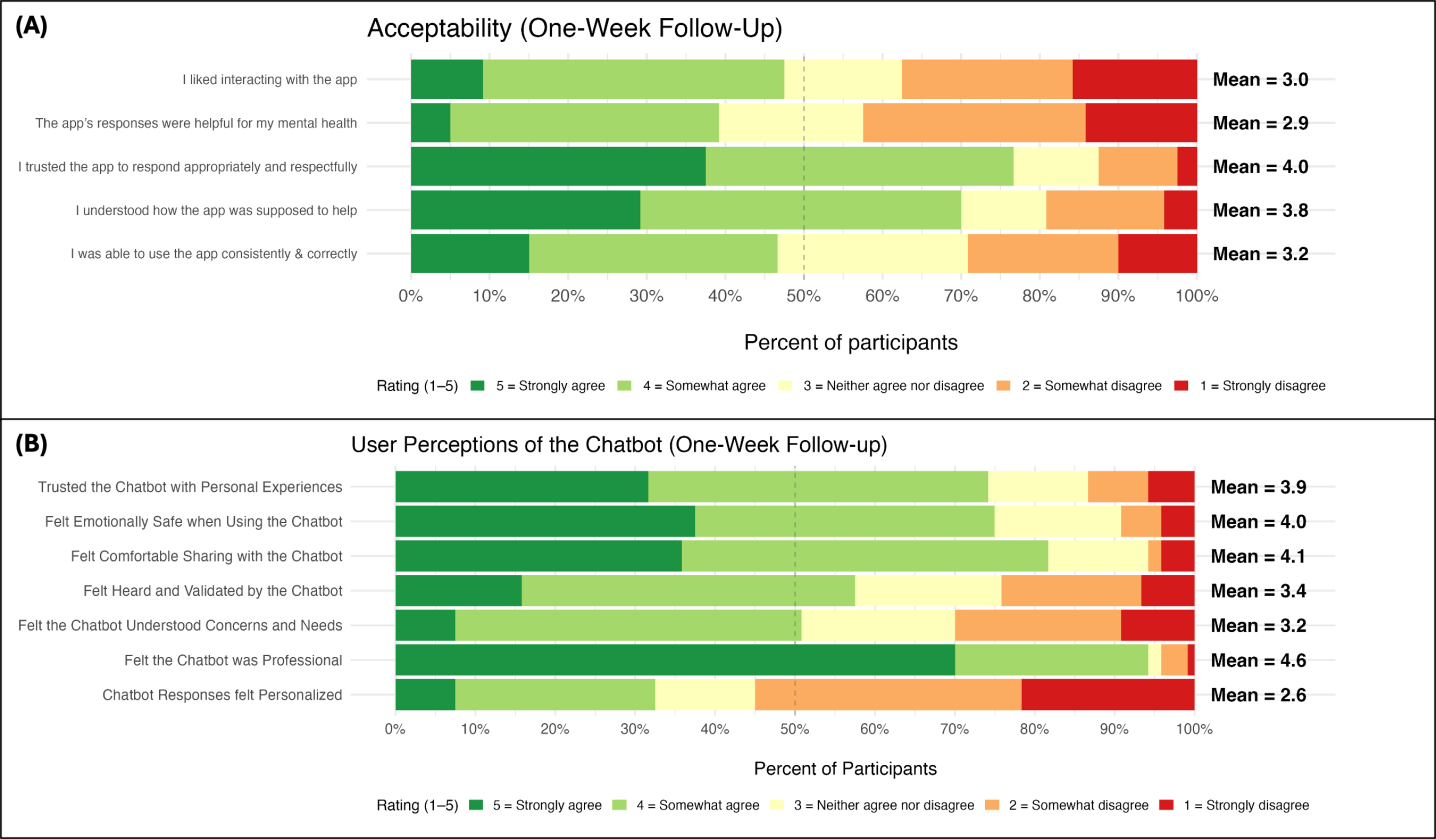
Panel (A) displays acceptability ratings of the chatbot; Panel (B) displays ratings of perceived advantages of the chatbot relative to traditional, human therapy.

Acceptability ratings at one week reflected a similar pattern of mixed strengths and areas to improve upon (Figure 2A). Most participants agreed or strongly agreed that they trusted the chatbot to respond appropriately and understood how the app was intended to help. However, broader indicators of acceptability were notably lower. Fewer than 10% strongly agreed that they liked interacting with the app, and fewer than half agreed or strongly agreed overall. Perceived helpfulness was also modest, with only ≈40% agreeing that the chatbot’s responses were helpful for their mental health. Ratings of self-efficacy also highlight an area for improvement, with fewer than half agreeing that they were able to use the app consistently and correctly. Household income was not related to first impression ratings (*r*=0.05, *P*=.59) or overall acceptability (*r*=–0.09, *P*=.37).

Therapeutic alliance ratings at 1-week follow-up were moderate across bond, task agreement, and goal agreement subdimensions, with mean item scores just narrowly above the scale midpoint (Figure S3 in Multimedia Appendix 1 [26-37]). Compared with human therapists, participants generally viewed the chatbot as more accessible, less stigmatizing, and easier to open up to and be honest with (Figure 2B). Taken together, these findings indicate that while the chatbot was generally viewed as safe, respectful, and understandable, with some key advantages over traditional therapy settings, participants’ experiences of enjoyment, perceived helpfulness, confidence in use, and personalization were more variable and less positive, on average. Household income was not related to perceived therapeutic alliance (*r*=−0.11, *P*=.28).

### Clinical Outcomes

#### Overview

Descriptive estimates of clinical outcomes at baseline and 2-week follow-up are shown in Table 1, and regression model results are shown in Table 2. Mixed-effects models showed a significant reduction in depressive symptoms from baseline to 2-week follow-up, corresponding to a small-to-moderate standardized effect size (Hedges *g*, computed using the baseline SD/Glass’s Δ=–0.32, 95% CI –0.52 to –0.11). Anxiety symptoms also decreased significantly, with a similar standardized effect magnitude (*g*=–0.32, 95% CI–0.50 to –0.14). Total-symptom models, including all participants with either PHQ-8 or GAD-7 scores >4, indicated significant overall improvement across the 2-week period (*g*=–0.26, 95% CI –0.42 to –0.09). Household income was not correlated with residualized change in total symptoms (*r*=–0.14, *P*=.16).

**Table 1.** Descriptive statistics for clinical outcomes at baseline and follow-up. The analytic sample for assessing depressive and anxiety symptoms included those with baseline patient health questionnaire (PHQ) or generalized anxiety disorder (GAD) scores >4, respectively. All other outcomes included participants with either PHQ or GAD >4 at baseline.

Measure/scale	Baseline		Two-week follow-up	
	N	Mean (SD)	N	Mean (SD)
Depressive symptoms (PHQ-8)	96	10.14 (4.10)	92	8.84 (4.39)
Anxiety symptoms (GAD-7)	107	10.38 (4.10)	102	9.18 (4.73)
Total symptoms (PHQ+ GAD)	116	18.85 (7.77)	111	16.94 (8.24)
Well-being (WHO-5)^a^	116	9.43 (4.34)	110	10.26 (4.82)
Psychological distress (K6)	116	7.38 (3.68)	110	6.59 (3.85)
Loneliness (UCLA-3)^b^	114	2.30 (1.72)	109	2.15 (1.99)
Chatbot attitudes (APOI^c^)	116	49.38 (9.34)	111	48.54 (11.02)

a WHO-5: World Health Organization-5 Well-Being Index.

b UCLA-3: University of California, Los Angeles 3-Item Loneliness Scale.

c APOI: Attitudes Towards Psychological Online Interventions.

**Table 2.** Longitudinal mixed-effects models predicting depressive, anxiety, and total symptom scores by time (baseline vs 2-week follow-up).

Covariate	Depressive symptoms (PHQ-8)^a^ (n=96)			Anxiety symptoms (GAD-7)^b^ (n=107)			Total symptoms (PHQ-ADS)^c^^d^ (n=116)		
	*β*	SE	*P* value	*β*	SE	*P* value	*β*	SE	*P* value
Time (follow-up vs baseline)	−1.30^e^	0.44^e^	.003^e^	−1.31^e^	0.38^e^	.001^e^	−2.01^e^	0.67^e^	.003^e^
Cohort 2 (vs cohort 1)	0.34	0.93	.718	−0.25	0.96	.79	−0.23	1.66	.89
Cohort 3 (vs cohort 1)	−0.31	0.89	.73	−1.1	0.93	.24	−2.11	1.61	.19
Age	0.00	0.04	.95	0.05	0.05	.29	0.05	0.07	.51
Woman (vs man)	−0.50	0.78	.52	0.11	0.82	.90	−0.93	1.43	.52
College degree (vs no degree)	−1.04	0.97	.28	0.3	1.01	.76	−1.52	1.77	.39
Postgraduate degree (vs no degree)	−1.82	1.08	.09	−1.92	1.11	.08	−4.25^e^	1.95^e^	.0^e^3
Unemployed (vs employed)	1.32	1.09	.22	-1.69	1.13	.14	−1.31	1.94	.50
Current student (vs employed)	−1.07	1.31	.41	-0.01	1.28	>.99	−1.32	2.14	.54
Asian/Asian American (vs White)	−1.09	1.19	.36	0.85	1.25	.50	0.04	2.14	.99
Black/African American (vs White)	2.30	1.28	.07	0.09	1.33	.94	1.75	2.17	.42
Multiple races (vs White)	1.68	1.78	.35	2.43	1.77	.17	6.22^e^	3.12^e^	.047^e^
Another race (vs White)	5.22	3.14	.10	2.75	3.29	.40	8.30	5.78	.15
Hispanic (vs Non-Hispanic)	4.79	2.74	.08	4.02	2.89	.16	9.88	5.18	.056
Interaction models^f^									
Time × baseline symptom Levels	−0.64^e^	0.17^e^	<.001^e^	−0.27^e^	0.09^e^	.001^e^	−0.35^e^	0.08^e^	<.001^e^

a Participants in the depressive symptoms model had to have a baseline PHQ score >4.

b Participants in the anxiety symptoms model had to have a baseline GAD score >4.

c Participants in the total symptoms model had to have either PHQ or GAD score >4 at baseline.

d PHQ-ADS: Patient Health Questionnaire Anxiety and Depression Scale.

e Statistically significant estimates.

f Interaction models were each tested separately, controlling for the same covariates as the main effects models above. Inverse interaction coefficients indicate that participants with higher baseline symptoms showed greater reductions from baseline to follow-up (ie, larger improvements).

#### Baseline Symptomatology as a Moderator

Across outcomes, participants with higher baseline symptom severity demonstrated larger improvements over the 2-week period, reflected by significant baseline-severity×time interactions (Table 2). To probe these interactions, we estimated the marginal intervention effect across a continuum of plausible baseline values. These estimates are model-based and reflect greater statistical uncertainty at the upper end of the baseline severity distribution, where observations were relatively sparse and should be interpreted cautiously.

**Figure 3.**
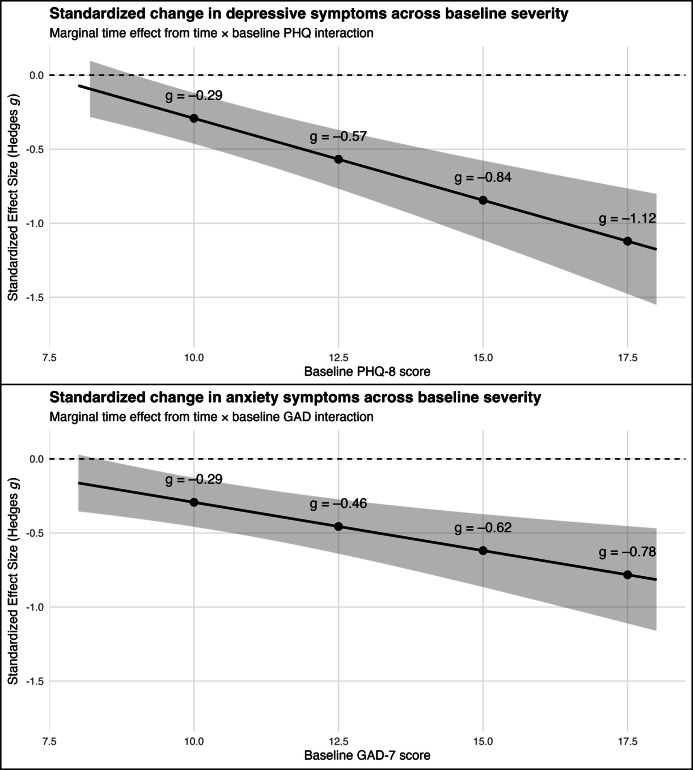
Curves show the model-estimated marginal effect of time (follow-up vs baseline) from mixed-effects models with a time× baseline interaction. Shaded regions indicate 95% CIs based on the delta method. GAD: generalized anxiety disorder; PHQ: patient health questionnaire.

For depressive symptoms, the magnitude of improvement increased steadily as baseline PHQ-8 severity increased (Figure 3A). At a baseline PHQ-8 score of 10, the predicted effect size was *g*=–0.29, consistent with a modest reduction. At baseline scores of 12.5 (*g*=–0.57) and 15 (*g*=–0.84), the corresponding predicted effects indicate medium to large improvements. At higher baseline severity (PHQ-8=17.5), the estimated improvement was *g*=–1.12, reflecting a very large effect. A similar pattern emerged for anxiety symptoms (Figure 3B). At a baseline GAD-7 score of 10, the predicted effect size was *g*=–0.29. This effect size increased at baseline scores of 12.5 (*g*=–0.46) and 15 (*g*=–0.62). At higher baseline severity (GAD-7=17.5), the model predicted a larger improvement (g=–0.78).

These marginal-effect curves show that while effect size magnitudes were modest at lower baseline symptom levels, participants with moderate to severe depressive or anxiety symptoms demonstrated substantially greater reductions over the 2-week exposure period. These results indicate that baseline severity meaningfully moderated clinical change, with higher-severity individuals showing the largest predicted reductions.

#### Secondary Clinical Outcomes

For secondary outcomes (Table 3), well-being increased (*g*=0.19, 95% CI 0.03-0.36) and psychological distress decreased (*g*=–0.21, 95% CI –0.38 to –0.04), with both effect sizes in the small-to-moderate range. Loneliness showed a small, nonsignificant reduction (*g*=–0.11, 95% CI –0.26 to 0.04). Attitudes toward mental wellness chatbots did not shift significantly across the exposure period, although the direction of change suggested slightly less favorable attitudes at follow-up (*g*=–0.09, 95% CI –0.24 to 0.05).

**Table 3.** Longitudinal mixed-effects models predicting secondary clinical outcomes by time (baseline vs 2-week follow-up). Participants in these models had to have either a patient health questionnaire (PHQ) or generalized anxiety disorder (GAD) score >4 at baseline.

Covariate	Wellbeing (WHO-5) (n=116)			Psychological distress (K6) (n=116)			Loneliness (UCLA-3)^a^ (n=116)			APOI^b^ – Therapy Chatbot (n=116)		
	*β*	SE	*P* value	*β*	SE	*P* value	*β*	SE	*P* value	*β*	SE	*P* value
Time (follow-up vs baseline)	0.85^c^	0.37^c^	.02^c^	−0.77^c^	0.32^c^	.02^c^	−0.19	0.13	.15	−0.88	0.68	.19
Cohort 2 (vs cohort 1)	−0.19	0.99	.84	−0.37	0.78	.64	−0.29	0.39	.46	3.32	2.18	.13
Cohort 3 (vs cohort 1)	0.31	0.96	.74	−0.25	0.76	.74	−0.55	0.38	.15	0.67	2.11	.75
Age	−0.04	0.04	.31	0.00	0.03	.89	0.01	0.02	.42	0.02	0.10	.82
Woman (vs man)	−1.07	0.85	.21	−1.17	0.67	.08	−0.27	0.34	.42	−0.17	1.88	.93
College degree (vs no degree)	−0.25	1.06	.81	−0.86	0.83	.30	−0.26	0.42	.54	−0.95	2.33	.68
Postgraduate degree (vs no degree)	0.79	1.16	.50	−1.80^c^	0.92^c^	.049^c^	−0.51	0.46	.27	−5.00	2.56	.05
Unemployed (vs employed)	−0.85	1.16	.46	−0.94	0.91	.31	0.10	0.45	.83	−0.38	2.54	.88
Current student (vs employed)	−1.00	1.27	.43	−0.35	1.00	.73	0.67	0.50	.18	−1.97	2.80	.48
Asian/Asian American (vs White)	0.33	1.27	.79	0.97	1.01	.33	0.64	0.50	.21	−0.31	2.81	.91
Black/African American (vs White)	0.47	1.29	.72	0.11	1.02	.91	1.13^c^	0.51^c^	.03^c^	1.86	2.84	.51
Multiple races (vs White)	−1.29	1.86	.49	2.39	1.47	.10	0.20	0.74	.79	−2.83	4.07	.49
Another race (vs White)	2.29	3.45	.51	4.85	2.72	.07	0.09	1.36	.95	9.29	7.57	.22
Hispanic (vs Non-Hispanic)	0.79	3.08	.80	5.21^c^	2.43^c^	.03^c^	1.12	1.22	.36	2.39	6.78	.72

a UCLA-3: University of California, Los Angeles 3-Item Loneliness Scale.

b APOI: Attitudes Towards Psychological Online Interventions.

c Statistically significant estimates.

#### Associations Between Usage and Symptom Change

Using residualized change scores adjusted for baseline symptom severity, Pearson correlations indicated that associations between chatbot usage metrics and total symptom change (PHQ-ADS) were small and not statistically significant (*r*=–0.13 to –0.08; Figure S4 in Multimedia Appendix 1 [26-37]). However, all correlations trended in the expected direction: participants who used the app more (more minutes, sessions, talk-turns, or days active) tended to show slightly greater symptom improvement. Given the structured, incentivized exposure window, variability in usage was intentionally constrained, limiting the ability to observe naturalistic dose–response associations.

#### Associations Between First Impressions and Symptom Change

Pearson correlations between first-impression ratings and residualized symptom change scores showed that 3 early experiential perceptions were significantly associated with greater improvement in total symptoms (PHQ-ADS; Figure S5 in Multimedia Appendix 1 [26-37]): feeling that the chatbot understood participants’ concerns and needs (*r*=–0.22, 95% CI –0.39 to −0.04), perceiving the chatbot as professional (*r*=–0.25, 95% CI –0.42 to –0.07), and rating the chatbot’s responses as personalized (*r*=–0.29, 95% CI –0.45 to –0.10). Correlations with emotional safety, trust, comfort sharing, and feeling heard or validated were small and nonsignificant.

### Experiential Predictors of Therapeutic Alliance and Acceptability

Exploratory models examined which specific experiences, assessed at the 1-week follow-up, were associated with therapeutic alliance and acceptability (Table 4). Although the trust item was removed due to multicollinearity issues (ie, Variance Inflation Factor >4), several experiences emerged as relevant correlates. Participants who felt more emotionally safe, heard/validated, and understood by the chatbot reported significantly higher therapeutic alliance scores and greater overall acceptability. Additionally, perceived personalization of the chatbot was significantly related to therapeutic alliance and acceptability, though with a slightly smaller effect. Taken together, participants’ sense of emotional safety and validation from the chatbot were the strongest experiential correlates of therapeutic alliance and acceptability.

These models also revealed notable demographic patterns. Relative to White participants, those identifying as Black, Hispanic, or multiracial/another race reported significantly higher acceptability, and Black participants also reported higher alliance. Additionally, participants in Cohort 2 (and for alliance, Cohort 3) reported higher ratings than those in Cohort 1, possibly reflecting iterations made to the chatbot based on the first cohort’s feedback.

**Table 4.** Experiential predictors of therapeutic alliance and acceptability scores. Participants in these models had to have either a patient health questionnaire (PHQ) or generalized anxiety disorder (GAD) score >4 at baseline. These 2 outcomes, therapeutic alliance and acceptability scores, are correlated *r*=0.81, *P*<.001.

Covariate	Therapeutic alliance			Acceptability scores		
	*β*	SE	*P* value	*β*	SE	*P* value
Felt emotionally safe using the chatbot	0.46^a^	0.18^a^	.01^a^	0.19^a^	0.08^a^	.02^a^
Felt comfortable sharing with the chatbot	−0.33	0.20	.10	0.00	0.09	.96
Felt heard and validated by the chatbot	0.64^a^	0.16^a^	<.001^a^	0.17^a^	0.07^a^	.02^a^
Felt the chatbot understood concerns and needs	0.38^a^	0.16^a^	.02^a^	0.29^a^	0.07^a^	<.001^a^
Felt the chatbot was professional	0.30	0.21	.15	0.08	0.09	.37
Felt the chatbot responses were personalized	0.26^a^	0.13^a^	.04^a^	0.12^a^	0.05^a^	.03^a^
Cohort 2 (vs cohort 1)	0.99^a^	0.31^a^	.002^a^	0.36^a^	0.14^a^	.009^a^
Cohort 3 (vs cohort 1)	0.97^a^	0.31^a^	.002^a^	0.21	0.13	.12
Age	0.00	0.01	.89	0.00	0.01	.53
Woman (vs man)	0.37	0.27	.16	0.03	0.12	.79
College degree (vs no degree)	−0.63	0.33	.06	−0.21	0.15	.14
Postgraduate degree (vs no degree)	−0.12	0.37	.75	−0.18	0.16	.26
Unemployed (vs employed)	0.48	0.37	.20	0.41^a^	0.16^a^	.01^a^
Current student (vs employed)	−0.77	0.43	.08	−0.20	0.19	.29
Asian/Asian American (vs White)	0.63	0.42	.13	0.16	0.18	.38
Black/African American (vs White)	1.20^a^	0.40^a^	.004^a^	0.44^a^	0.18^a^	.01^a^
Multiple races (vs White)	1.05	0.57	.07	0.54^a^	0.25^a^	.03^a^
Another race (vs White)	1.52	1.05	.15	1.00^a^	0.46^a^	.03^a^
Hispanic (vs Non-Hispanic)	1.21	0.93	.20	1.09^a^	0.41^a^	.009^a^

a Statistically significant estimates.

## Discussion

### Summary of Findings

This mixed methods pilot evaluated (1) user experience and acceptability, (2) feasibility of safety monitoring, and (3) an exploratory clinical signal for an early-stage generative AI mental wellness chatbot. Under an incentivized, time-limited protocol, participants reported the chatbot as accessible and emotionally validating while noting limitations in personalization and conversational depth. Safety workflows were feasible: 8% of participants triggered an automated safety alert, all were reviewed within 2 hours, and none required outreach under prespecified criteria. Models showed small-to-moderate pre–post reductions in depressive and anxiety symptoms, with larger predicted improvements among participants with higher baseline severity, though model-predicted effects at the upper end should be interpreted cautiously given sparser data among those with elevated baseline symptoms. Together, findings support procedural feasibility and identify candidate therapeutic targets to inform ongoing refinement and future controlled trials.

### Interpretation and Comparison With Prior Work

#### Clinical Signal

Participants demonstrated measurable improvements in depressive and anxiety symptoms over the 2-week exposure period, despite the brief, incentivized format and the early developmental stage of the prototype. Small-to-moderate reductions were also observed for psychological distress and well-being, suggesting that even limited engagement with the chatbot produced detectable short-term clinical benefits. These findings are notable given the relatively mild symptom severity of the enrolled sample and the modest exposure (≈60 min per week).

Baseline symptomatology moderation analyses provide critical context for understanding these effects in comparison to other recently published chatbot studies. Participants with more severe baseline symptoms experienced substantially greater improvement, with standardized effect sizes increasing sharply with more elevated baseline severity. This pattern indicates that the chatbot prototype’s clinical impact is not uniform but may vary as a function of initial symptom burden; however, given the restricted risk profile of this pilot sample, moderation patterns should be interpreted cautiously, especially at the higher end, given sparse data and wider CIs. Indeed, the modest average effects observed in the full sample are partly a function of the sample’s relatively low baseline severity, not necessarily a limitation of the chatbot itself.

This has direct relevance for interpreting our findings within the burgeoning literature on mental wellness chatbots. Recent trials of more mature systems, such as Therabot [9] and Ash [44], have reported larger effect sizes than the main effects estimated herein. However, these trials deliberately recruited participants with moderate to severe symptoms, required higher-frequency, longer-duration exposure, and used different statistical modeling approaches to estimate effect sizes. Because symptom severity is a strong determinant of effect magnitude, studies that enroll more symptomatic participants naturally provide more opportunity for measurable change [45]. Our moderation results reinforce this point as higher baseline symptoms were associated with substantially larger predicted improvements. Thus, while direct comparisons to more established chatbots are not yet appropriate given differences in product maturity, engagement requirements, and sample symptom levels, our findings suggest that this early-stage prototype has the potential to produce clinically meaningful effects when used by individuals with greater initial symptom severity. We interpret this evidence as proof of concept, clarifying both the potential of the nascent system and the likely improvement as the product becomes more refined and is tested in samples with higher baseline symptomatology.

#### User Experience and Design Considerations

The convergent quantitative and qualitative findings highlight both the prototype’s strengths and the most critical areas for refinement. Participants frequently characterized the chatbot as accessible, nonjudgmental, and easy to use. These positive qualities strongly aligned with the goal of AI-supported mental health tools to lower barriers to care [46]. Users’ impressions also aligned with high usability ratings and moderate therapeutic alliance scores, indicating that participants were able to form a basic sense of responsiveness and support with the tool. Importantly, alliance in AI-mediated interactions is conceptually distinct from the relational bond cultivated with human therapists and should not be interpreted using the same benchmarks [47]. As others in this area have argued [48], alliance plays a more modest and indirect mechanistic role in digital interventions. In this context, the moderate alliance observed presently likely represents a realistic upper bound for early-stage prototypes, but is nevertheless a key focus for forthcoming product development.

A core design tradeoff in AI tools is that stronger safety and content-guardrail constraints often limit the natural fluidity of conversation, shaping how users experience and evaluate the system. Integration of the qualitative and quantitative findings revealed consistent limitations in personalization, conversational depth, and perceived helpfulness. Some participants described the interaction pattern as repetitive, formulaic, or context-insensitive, which directly aligns with lower quantitative ratings on items related to personalization and understanding. Acceptability ratings also revealed mixed experiences: trust and perceived appropriateness were strong, whereas enjoyment, perceived helpfulness, and self-efficacy in using the chatbot correctly were more variable. Notably, attitudes toward mental wellness chatbots overall became slightly less favorable from baseline to follow-up, suggesting that initial optimism or curiosity may have surpassed the practical realities of interacting with an early-stage prototype, which is a pattern frequently reported in digital mental health when user expectations exceed current product capabilities [49,50].

Participants often expressed expecting immediate advice capable of resolving distress within a single interaction. Although understandable, such expectations run counter to core therapeutic principles. Evidence-based psychotherapy discourages providing directive advice at the outset of treatment, before adequate case understanding has been established [51], and even the most effective interventions require ongoing practice and reflection rather than instant resolution. The present mental wellness chatbot emphasizes clarifying questions and reflective prompts that can feel repetitive or circular but are designed to mirror therapy processes [25]. In contrast, general-purpose AI systems (ie, ChatGPT) often provide rapid, directive answers that may feel more responsive yet do not align with therapeutic process fidelity. When user expectations for immediate relief exceed what a clinically guided system can reasonably provide, perceived helpfulness may decline, emphasizing the need to set clear expectations about the capabilities and purpose of early-stage mental wellness chatbots.

Unexpectedly, demographic patterns also offer important implications for chatbot-based mental well-being tools. Black, Hispanic, and multiracial participants reported higher acceptability and alliance than White participants. These findings are exploratory and should be interpreted cautiously, particularly given sample size considerations and the structured, incentivized design of the study. While chatbot-based interactions may represent a potentially lower-barrier modality for some groups who face disproportionate barriers in traditional mental health systems, further investigation in larger and more diverse samples under naturalistic conditions is required before drawing conclusions regarding equity implications.

#### Therapeutic Signal and Early Mechanisms of Change

Consistent with theory on early therapeutic processes, several first-impression ratings, including feeling understood, perceiving professionalism, and experiencing personalization, were significantly associated with greater symptom improvement. These associations are consistent with participant narratives describing moments of emotional validation and goal-setting support. Importantly, these associations emerged despite the prototype’s limited personalization, suggesting that even brief experiences of attunement or perceived competence may exert disproportionate influence on therapeutic expectancies and engagement.

Conversely, the absence of dose–response relationships between usage metrics and symptom change is likely attributable to the structure of the pilot study itself. Engagement was incentivized, compressed, and highly uniform across participants, limiting the natural variability needed to detect behavioral predictors of improvement. Qualitative reports were consistent with this interpretation: while some participants sought emotional support, others acknowledged using the app primarily to meet compensation thresholds. Longer, more naturalistic trials (ie, flexible use) are likely to provide a more accurate test of organic engagement–outcome relationships.

#### Safety Monitoring

Finally, the safety monitoring procedures tested in this pilot support the feasibility of this clinician-supervised mental wellness chatbot. In this study, 8% of participants triggered at least one automated safety alert, and all alerts were promptly reviewed by a clinician. Consistent with the deliberately high-sensitivity safety classifier and nonacute sample, none met the prespecified threshold for phone-based outreach (eg, acute or imminent risk). This pattern suggests that real-time safety guardrails can successfully surface potentially concerning content while keeping the volume of urgent follow-ups manageable, which is encouraging for larger human-in-the-loop trials. Nonetheless, further testing among higher-risk samples with longer exposure periods is needed to determine how the workload associated with safety monitoring would differ when higher-risk users (eg, those with suicidal thinking) are not excluded.

### Implications for Early-Stage Development of Mental Well-Being Chatbots

Collectively, these mixed methods findings suggest that even a minimally refined generative AI–based chatbot can deliver a safe and accessible user experience that may be associated with modest short-term improvements in mental health symptoms under pilot conditions. The iterative cohort design highlights the value of rapid user feedback loops for refining conversational flow, session pacing, and safety monitoring protocols. As LLM-based systems continue to evolve, improvements in contextual coherence, personalization, and adaptive responsiveness may strengthen user experience and engagement, but these possibilities require evaluation under more rigorous, controlled conditions.

As chatbot systems are iteratively refined, tracking theory-consistent mediators, such as experiential and behavioral change processes, across product versions will be critical to determining whether design modifications meaningfully enhance user experience and clinical signal. Longer-term randomized studies under more naturalistic conditions are also needed to evaluate durability and to examine how chatbots may operate within integrated care pathways; for example, as a pretherapy intake tool, a between-session reinforcement, a maintenance tool following treatment, or a monitored entry point that escalates to human clinicians when symptom trajectories fail to improve or deteriorate [52]. Finally, future work should continue to explore the balance between usability and therapeutic rigor, as highly engaging conversational interfaces must also preserve core therapeutic processes that may not always prioritize immediate gratification.

### Limitations

Regarding limitations, the brief, 2-week exposure period limits conclusions about durability, particularly because outcomes were measured during or immediately after active use. Such designs may capture proximal engagement effects rather than sustained change [14]. However, this timeframe was appropriate for a pilot focused on usability, acceptability, and early clinical signals, and it enabled rapid prototype iteration. The single-arm pre–post design also raises the possibility of regression to the mean. In the absence of a randomized comparison group, improvements cannot be attributed definitively to the chatbot intervention, yet the magnitude of observed improvements—especially at higher baseline severity—appears larger than short-term changes typically reported in waitlist-controlled meta-analyses [53,54]

Participants were recruited from a convenience panel familiar with providing user-experience feedback on digital apps (dscout), which may limit generalizability, but provided high compliance and rich qualitative feedback appropriate for early-stage evaluation. Relatedly, pilot sampling purposefully excluded individuals with acute risk and those currently in treatment, consistent with the nascent state of this chatbot app. Engagement during this pilot was supported by usage-contingent incentives to ensure adequate exposure for prototype evaluation. This structure was appropriate for assessing usability, safety monitoring procedures, and early experiential processes, but engagement metrics should be interpreted as reflecting the feasibility of the study protocol rather than voluntary real-world uptake. While such incentives are common in early prototyping research, future effectiveness trials should evaluate voluntary, real-world engagement. Moreover, incentive structures may have influenced not only engagement patterns but also self-reported experiential and clinical outcomes, introducing potential response bias and limiting the internal validity of these self-reported measures. Although exploratory analyses did not detect significant associations between household income and self-reported experiential or clinical outcomes, income data were missing for 13.6% of participants, which may introduce selection bias if nonresponse was systematic.

Finally, the qualitative aspects relied on LLM-assisted content analysis to identify recurring experiential patterns rather than on researcher-led reflexive thematic analysis. Although theme stability was assessed across multiple independent model runs and outputs were reviewed for coherence, this approach did not incorporate traditional qualitative features such as prolonged coding immersion, formal reflexivity procedures, or independent human coding. Accordingly, qualitative findings should be interpreted as descriptive and exploratory, appropriate to the formative aims of this pilot study.

### Conclusions

This pilot user experience study establishes critical feasibility and proof of concept for an early-stage mental well-being chatbot that can deliver meaningful and safe usability and measurable short-term symptom improvement, even in a mildly symptomatic sample. Transparent documentation of iterative development, combined with mixed methods insights and severity-based marginal effects, highlights both promising preliminary findings and the design targets most likely to enhance future engagement and clinical impact. These findings guide continued refinement and set the stage for rigorous, longer-term trials evaluating sustained engagement, clinical durability, and performance in more symptomatic samples.

Beyond this prototype, the study demonstrates a practical evaluation template for early-stage LLM mental health tools that combines user experience, safety workflow feasibility, and exploratory clinical outcomes with explicit caution about efficacy inference. As mental wellness chatbots continue to develop, transparent reporting of design constraints, safety monitoring, and user-perceived therapeutic processes will be critical for responsible iteration and for interpreting early clinical signals prior to larger randomized trials and real-world deployment.

## Supplementary material

10.2196/90644 Multimedia Appendix 1 Additional figures and information.

10.2196/90644 Checklist 1 CONSORT checklist.

## Abbreviations

AI: artificial intelligence
CONSORT: Consolidated Standards of Reporting Trials
GAD: generalized anxiety disorder
IUS: Intervention Usability Scale
LLM: large language model
PHQ: patient health questionnaire
PHQ-ADS: Patient Health Questionnaire Anxiety and Depression Scale
