# Supplementary material for: User Experience and Early Clinical Outcomes of a Mental Wellness Chatbot for Depression and Anxiety: Pilot Evaluation Mixed Methods Study
Source: JMIR Form Res. 2026 Apr 14;10:e90644. doi: 10.2196/90644 (PMC13094381; doi:10.2196/90644)
Supplement: Multimedia Appendix 1 [file formative-v10-e90644-s001.docx]

**Supplemental Materials**

**AI Architecture**

The app employed a modular architecture integrating several connected AI components, powered collectively by numerous LLM prompts and orchestrated workflows. Beginning in Cohort 2, and based on user feedback, each session (e.g., following a new login after ≥12 hours) started with participants selecting their preferred engagement duration (see Figure S1). Cohort 1 did not include timed sessions, which users identified as a limitation that was subsequently addressed. Across all versions, the Main-Conversation agent served as the central conversational node. This agent maintained advanced long-term memory, supported multimodal input/output, and stored fully deidentified information for continuity across sessions. Authentication and user access were managed through a secure industry-standard authentication service. LLM workflows were built using LangChain, with LangSmith providing tracing, debugging, and evaluation for the multi-agent architecture. At the end of each session, a Summary-Agent generated a recap that was stored as persistent memory for future interactions. At the time of writing, both conversational and summarization tasks were powered by the OpenAI API (GPT-4o or GPT-4o-mini). Exact prompts, system instructions, and model parameters are proprietary and cannot be publicly disclosed. Additional screenshots of the interface are shown at the end of the Supplement (Figures S6 – S8).

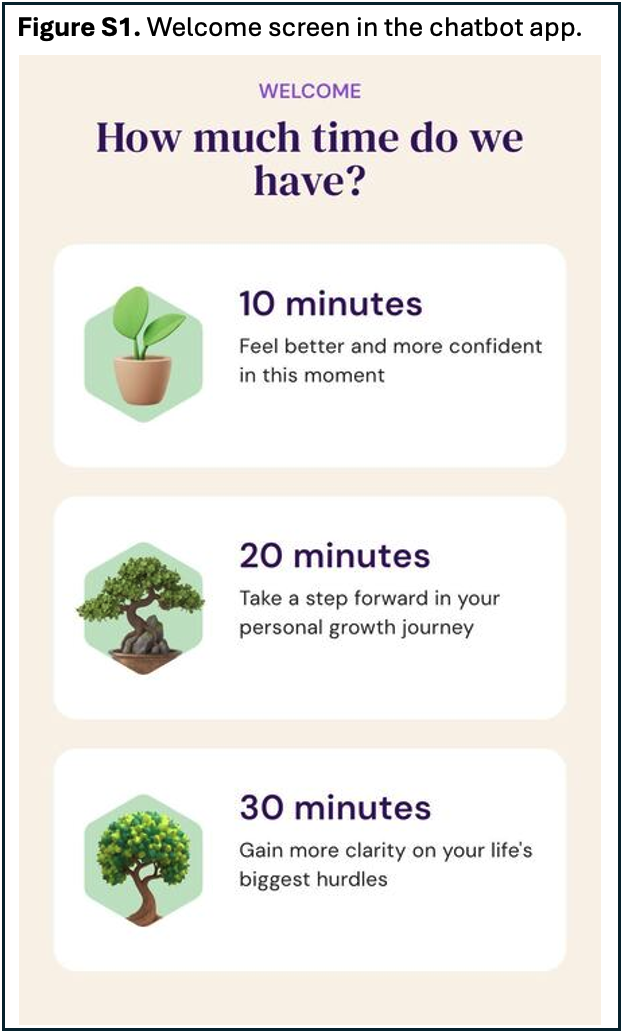


**Therapeutic Framework and Design Intent.** The chatbot was designed to simulate early-session processes common to supportive and cognitive-behavioral therapy (CBT), rather than to deliver structured, manualized treatment protocols. The guiding therapeutic principles included (a) emotional validation and reflective listening, (b) collaborative goal clarification, and (c) brief coping strategy support (e.g., grounding, cognitive reframing prompts, behavioral activation suggestions).

Sessions were semi-structured. Upon login, users selected a preferred session length. The conversational agent then initiated an open-ended emotional check-in (e.g., “What’s been on your mind lately?”), followed by reflective summarization and validation of expressed emotions. When appropriate, the system prompted users toward small, actionable steps (e.g., identifying one manageable goal for the day) or offered brief skills-based exercises (e.g., paced breathing, perspective-taking questions). The system was designed to prioritize clarification and reflective questioning before offering suggestions, in alignment with supportive therapy principles.

While responses were generated by a large language model, they were constrained by structured system-level instructions emphasizing emotional safety, nonjudgment, and non-directiveness. The chatbot was explicitly restricted from providing diagnoses or prescriptive medical advice. Thus, therapeutic techniques (e.g., validation, goal-setting prompts) reflect intentional design scaffolding rather than purely emergent behavior.

**Hypothesized and Targeted Mechanisms**. The hypothesized early mechanisms of change centered on common supportive and cognitive-behavioral therapy processes. Specifically, emotionally validating responses were intended to increase perceived attunement and psychological safety; collaborative clarification of goals was designed to enhance self-efficacy and agency; and brief coping or activation prompts were aimed at producing short-term reductions in distress. Together, these proximal processes were expected to support engagement and facilitate modest short-term symptom improvement within the context of this pilot-stage evaluation. Examples of the chatbot’s response patterns included reflective validation (e.g., “It sounds like you’ve been feeling overwhelmed and exhausted. That makes sense given everything you described.”), clarification prompts to deepen self-reflection (e.g., “When you say you feel stuck, what does that look like in your day-to-day routine?”), and gentle activation suggestions (e.g., “Would it feel manageable to try one small step today, even something as simple as stepping outside for five minutes?”). Exact prompts, internal system instructions, and model parameters remain proprietary and are not publicly disclosed.

The chatbot’s targeted mechanisms/processes align with the experiential outcomes examined in the acceptability and user experience analyses (e.g., perceived understanding, personalization, and emotional safety), providing conceptual continuity between intervention design and measured outcomes. In other words, the study measured these very processes that the chatbot was designed to target. Observed associations between these experiential variables and symptom change were interpreted as preliminary mechanistic signals, informing iterative refinement of the prototype and guiding design considerations for subsequent controlled trials.

**Safety Classifier and Monitoring.** Within the chatbot app**,** a continuous (real-time), automated LLM-based safety classifier monitors for potential high-risk content from the user (including risk to self, risk to other and risk from others). The real-time safety classifier was calibrated for high sensitivity during this early stage of chatbot testing. When the real-time safety classifier detects potentially high-risk content, participants are provided 24/7 crisis and emergency resources (e.g., 988, 911) right away by the chatbot and notified that a human clinician from the study team may reach out to them within the next day. During the study, all transcript-based safety alerts (i.e., any time the classifier detected potential high-risk content from the user) were reviewed by a study clinician within two hours to determine whether phone-based outreach was indicated. The study protocol was to conduct phone-based outreach for a safety check and potential emergency intervention if the transcript contained expressions of suicidal thinking or potential acute or imminent risk of harm to self, to others, or from others. A study clinician also conducted phone-based outreach for a safety check within 24 hours of any two-week follow-up survey responses indicating recent active suicidal thoughts, plan, or intent on the Columbia Suicide Severity Rating Scale (C-SSRS) [1], which was triggered only if participants scored ≥1 on the PHQ-9 ninth item at follow-up. The chatbot also includes other safeguards to promote user safety, ethical integrity, and responsible AI behavior. For example, the chatbot is explicitly restricted from providing clinical diagnoses, prescribing medication, or offering advice that could encourage self-harm or risky behaviors. It uses structured refusal patterns and fallback messages that direct users to emergency or human-supported resources when high-risk content is detected. Prior to launch, the chatbot underwent multi-round red-teaming by external AI safety experts and internal clinical reviewers to identify and mitigate potentially harmful or biased outputs. The system was also tested against synthetic “stress-case” conversations generated via GPT-based adversarial prompting to evaluate its crisis-response boundaries and escalation accuracy.  **Participants and Procedures Supplement**

Participants were recruited between September and November 2025 through dscout (dscout Inc., Chicago, IL; https://www.dscout.com), an online research platform that maintains a large opt-in panel of adults across the United States who volunteer for behavioral and user-experience research. The platform enables targeted recruitment based on demographic and screening criteria and has been used in prior digital mental-health studies. All screening and data collection occurred through the dscout mobile application and secure web interface. Prospective participants completed an electronic consent form followed by a brief screening survey to determine eligibility.
 Eligible participants were adults aged 21 years or older who resided in the United States but outside of Washington, Nevada, Vermont, California, Colorado, Connecticut, and Virginia. These exclusions reflected privacy and data-handling restrictions under state-specific regulations and Terms & Conditions. To qualify, participants were required to report at least mild symptoms of anxiety or depression, defined as a score of 3 or higher on either the two-item Patient Health Questionnaire (PHQ-2) or two-item Generalized Anxiety Disorder scale (GAD-2) [2]. Participants prescribed psychotropic medication were eligible if their medication regimen had remained stable for at least three months. To ensure participants’ willingness to engage meaningfully with the chatbot, individuals were also required to score 5 or higher on a single item assessing openness to engaging with a digital tool to improve mental health (“On a scale of 0–10, how open are you to engaging with something that might help improve your mental health or emotional well-being?”). Participants needed access to an iPhone with web-enabled device compatible with the study platform. The apple operating system was required because the chatbot app was only able to programmed for iOS.
 Individuals were excluded if they endorsed any self-harm thoughts on item 9 of the PHQ-9 (“Thoughts that you would be better off dead, or of hurting yourself in some way?”). Those excluded for potential suicidal ideation were automatically provided with extensive crisis resources, including the 988 Suicide and Crisis Lifeline, Crisis Text Line, and local emergency options. Additional exclusion criteria included self-reported serious mental illness or psychotic symptoms (e.g., schizophrenia spectrum or bipolar disorders), current participation in psychotherapy or counseling, and self-reported high-risk levels of alcohol or drug use.
 Screening data were automatically scored within dscout, but a key limitation of the platform was that dscout could not compute summed PHQ and GAD scores in real time. As a result, PHQ-2 and GAD-2 scores were manually calculated after data export. Of the 3,406 individuals who completed the screening survey, 688 (20.2%) progressed to this manual exclusion step. Among these 688, 504 (73.3%) were excluded for not meeting the minimum symptom severity threshold (PHQ-2 or GAD-2 score ≥ 3), leaving 184 participants (~5% of all screeners) eligible. See Participant Flow Diagram in the figure below.
 The target sample was 40 participants in each of three cohorts (120 total). To promote demographic diversity and generalizability, the sample was balanced such that no more than 65% of participants identified as women and no more than 65% as non-Hispanic White. Aside from oversampling male and non-White participants to improve representativeness, invitations were issued at random from the eligible pool. A total of 137 eligible participants were invited but, because not all eligible participants ultimately downloaded the app or completed onboarding, 125 participants in total were enrolled. Across the full screening process, the most common exclusion reasons were currently being engaged in mental health treatment (n = 833, ~24%), reporting a serious mental illness diagnosis or psychosis-like symptoms (n = 545, ~16%), and endorsing thoughts of self-harm above 0 on the ninth item of the PHQ (n = 84, ~2.5%). We note that these relatively high rates of mental health concerns likely reflect both (a) self-selection of individuals with elevated mental health needs into a mental health-focused study, and (b) thep possibility that some participants responded in ways they believed would increase their chances of being selected.

**Survey Measures**

**Primary Clinical Outcomes.** All clinical outcomes were assessed at both baseline and the two-week follow-up survey. Depressive symptoms were measured using the first eight items of the nine-item Patient Health Questionnaire (PHQ-9 [3]. Item 9 (“Thoughts that you would be better off dead or of hurting yourself in some way”) was excluded from baseline as this item was asked in the screener and all participants scored a 0 on this, as part of eligibility criteria. The remaining eight items were rated on a 4-point Likert scale ranging from 0 (“not at all”) to 3 (“nearly every day”) and summed to yield scores from 0 to 24, with higher values indicating greater depressive symptom severity. Prior work confirms that the shortened 8-item version of this scale has similar sensitivity to the full PHQ-9 [4]. Internal consistency in the present sample was α = 0.81 at baseline and α = 0.80 at two-week follow-up.


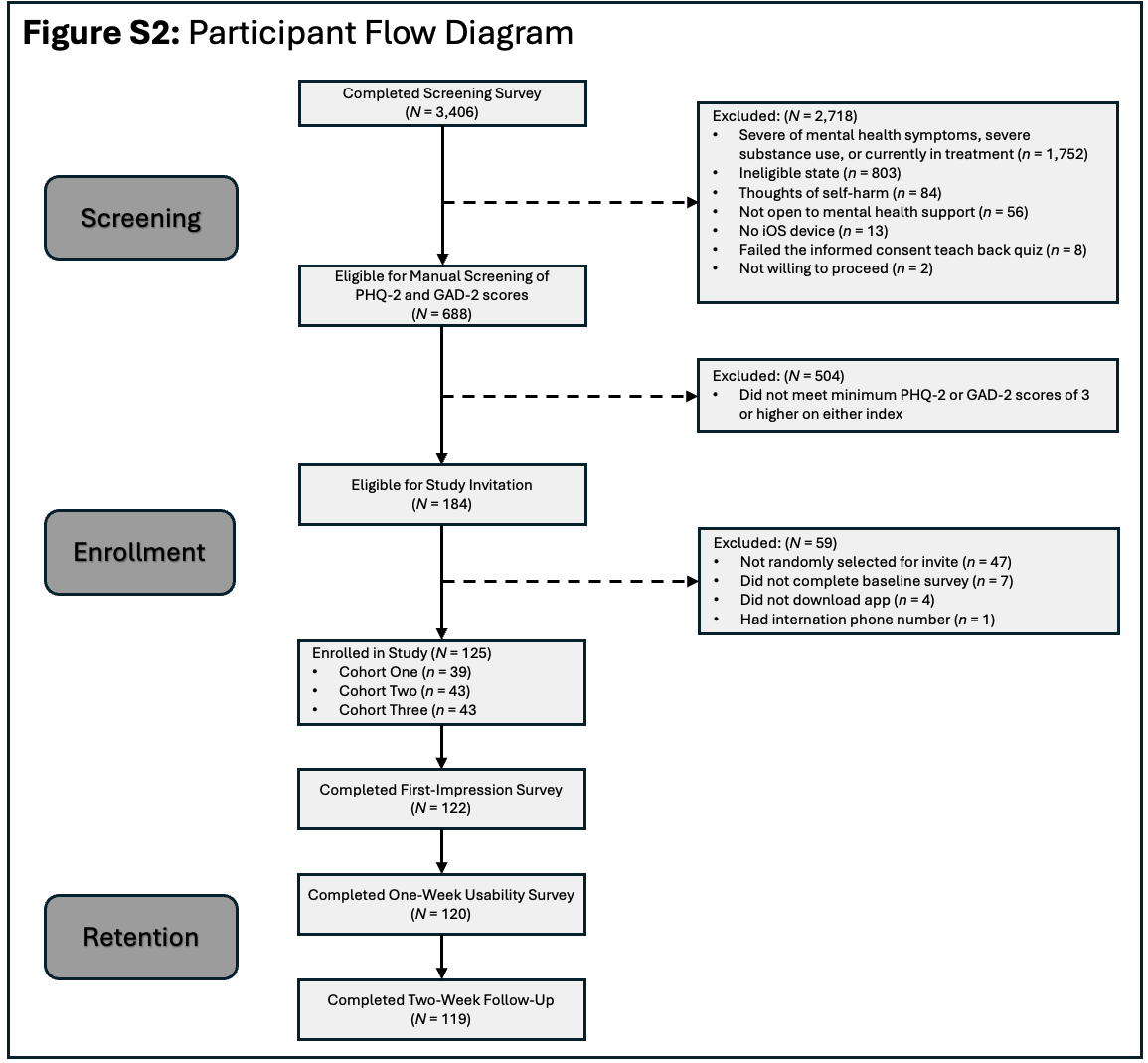
Anxiety symptoms were measured using the seven-item Generalized Anxiety Disorder scale (GAD-7) [5], which assesses the frequency of core anxiety symptoms over the past two weeks on the same 0–3 response scale. Scores range from 0 to 21, with higher scores indicating greater anxiety severity (α = 0.83 at baseline; α = 0.87 at follow-up).
 To index overall internalizing symptom burden, PHQ-8 and GAD-7 scores were combined to create the Patient Health Questionnaire–Anxiety and Depression Scale (PHQ-ADS), a validated composite measure of depression and anxiety severity [6]. The PHQ-ADS reflects shared variance in depressive and anxious affect and has demonstrated strong psychometric properties and responsiveness in clinical research. In the present pilot, the composite was included to facilitate interpretation of global symptom change rather than to designate a distinct confirmatory primary endpoint (α = 0.87 at baseline; α = 0.88 at follow-up).

 **Secondary Clinical Outcomes.** Positive well-being was assessed using the World Health Organization–Five Well-Being Index (WHO-5) [7], which consists of five positively worded items (e.g., “I have felt cheerful and in good spirits”) rated on a 6-point scale from 0 (“at no time”) to 5 (“all of the time”). Scores are summed yielding a range from 0 to 25, with higher scores reflecting greater subjective well-being (α = 0.82 at baseline; α = 0.85 at follow-up).
 General psychological distress was measured using the Kessler Psychological Distress Scale (K6) [8]. The K6 includes six items assessing nonspecific distress symptoms (e.g., feeling nervous, hopeless, restless, or worthless), rated from 0 (“none of the time”) to 4 (“all of the time”), yielding total scores from 0 to 24. Higher scores indicate greater psychological distress (α = 0.74 at baseline; 0.80 at follow-up).
 Loneliness was assessed with the UCLA Loneliness Scale–Short Form [9], a three-item measure adapted from the original 20-item UCLA Loneliness Scale. Items are rated on a 4-point scale from 1 (“never”) to 4 (“often”), with higher scores reflecting greater perceived loneliness (α = 0.73 at baseline; α = 0.83 at follow-up).
 Attitudes toward digital mental-health tools were measured using the Attitudes Toward Psychological Online Interventions questionnaire (APOI) [10], adapted for mental wellness chatbots by substituting references to “online therapy” with “therapeutic chatbot.” The APOI contains 16 items rated on a 5-point Likert scale from 1 (“strongly disagree”) to 5 (“strongly agree”). Items were scored so that higher values reflect more favorable attitudes toward mental wellness chatbots, and a total APOI score was computed as the mean of all 16 items (α = 0.88 at both baseline and follow-up).

**Therapeutic Alliance.** Therapeutic alliance was measured at the one-week follow-up using three items adapted from the Working Alliance Inventory bond, goal, and task components. Each item was rated on a 1–10 scale, with anchors of 1 (“not at all true”), 5 (“undecided”), and 10 (“completely true”). Items assessed (a) perceived bond (“I have a good working relationship with the chatbot”), (b) agreement on goals (“I understand and agree with the goals the chatbot has set for me or that I’ve set with the chatbot”), and (c) agreement on tasks (“The chatbot and I agree on the best approach for addressing my problems”). Responses were averaged to yield a total alliance score, with higher values indicating stronger working alliance (α = 0.88).

**Acceptability.** Acceptability of the chatbot was assessed at the one-week follow-up using five items grounded in the Theoretical Framework of Acceptability for healthcare interventions (TFA) [11]. Items were rated on a 1–5 scale (1 = “strongly disagree,” 5 = “strongly agree”) and assessed affective attitude (“I liked interacting with the app”), perceived effectiveness (“The app’s responses were helpful for my mental health”), ethicality/trust (“I trusted that the app would respond appropriately and respectfully”), intervention coherence (“I understood how the app was supposed to help my mental health”), and self-efficacy (“I felt able to use the app consistently and in the right ways to support my mental health”). Responses were averaged to yield a total acceptability score, with higher values indicating greater acceptability (α = 0.86).

**Usability.** Usability was assessed at the one-week follow-up using the Intervention Usability Scale (IUS) [12], an adaptation of the System Usability Scale for psychosocial and digital-mental health interventions. Items were rated on a 1–5 scale (1 = “strongly disagree,” 5 = “strongly agree”) and included positively- and negatively-worded statements about frequency of use, complexity, ease of use, consistency, learnability, and confidence. Standardized scoring procedures were applied as instructed by the scale developers (positive items: score − 1; negative items: 5 − score) and results were multiplied by 2.5 to yield a 0–100 usability index (higher = better usability). Internal consistency in this study was α = 0.79.

**Chatbot User Perception Items.** Participants rated their experiences with the chatbot during the first-impression and one-week follow-up survey using seven single-item indicators, each scored on a 1–5 Likert scale (1 = “strongly disagree,” 5 = “strongly agree”). Items assessed perceived trust (“Trusted the chatbot with personal experiences”), emotional safety (“Felt emotionally safe when using the chatbot”), comfort with disclosure (“Felt comfortable sharing with the chatbot”), perceived empathy (“Felt heard and validated by the chatbot”), perceived understanding (“Felt the chatbot understood concerns and needs”), professionalism (“Felt the chatbot was professional”), and personalization (“Chatbot responses felt personalized”). Higher scores on each item reflect more positive experiential perceptions of the chatbot.

 **Perceived Advantages Compared to Human Therapists.** At the one-week follow-up, participants rated several potential advantages of interacting with the chatbot compared to a human therapist. Knowing that not all participants have experienced human therapy, the stem instructed participants to think about what it might be like even if they had no prior experience. Items were rated on a 1–5 scale (1 = “strongly disagree,” 5 = “strongly agree”). For the present analyses, we focused on six key perceived advantages: neutrality (“The chatbot was more neutral and objective”), immediacy of openness (“The chatbot made it easier to open up right away”), honesty (“It was easier to be honest with the chatbot”), reduced fear of judgment (“I felt less judged when talking to the chatbot”), availability (“The chatbot was more available when needed”), and ease of disclosure (“It was easier to express what I really think or feel when talking to a chatbot”). Higher values indicate stronger endorsement of each perceived advantage.

**Open-Ended Qualitative Items**

Sets of qualitative prompts were administered immediately after the first impression survey and the one-week follow-up survey. Most responses were typed text; each survey also included one selfie-style video prompt (2 minutes) that was automatically transcribed and analyzed as text. All qualitative responses were used to characterize participants’ initial reactions, evolving perceptions, sources of confusion or surprise, perceived benefits and limitations, and suggestions for improvement.

**First Impression Questions.** Participants completed the following open-ended prompts immediately after their first session using the chatbot:

1. What were your thoughts or how did it make you feel when you learned the app was a therapy chatbot and not human therapy?
2. Thinking back to the introduction of the app, what was confusing or unclear?
3. What information do you feel was missing from the setup or onboarding that would help you feel more prepared to use the chatbot?
4. Before you used the chatbot, what did you expect the experience would be like? Did anything surprise you?
5. [Video, up to 2 minutes]: In a selfie-style video, what was your experience like the first time you used the chatbot? Please answer thoughtfully and clearly.
6. After using the chatbot for the first time, what, if anything, was confusing, frustrating, or unexpectedly helpful?
7. If you weren’t part of a paid study, what about your first experience would make you want to keep using the app? And, what might deter you?
8. Please share any other thoughts or reactions about your first experience that you haven’t had a chance to mention yet.

**One-Week Follow-Up Questions.** Participants completed the following open-ended prompts immediately after the one-week survey:

1. [Video, up to 2 minutes]: What is your overall impression of your experience with the chatbot app? What stood out to you, both positively and negatively?
2. Some people take time to get used to talking to a chatbot, while others feel comfortable right away. In your experience, what was it like getting started and getting used to it?
3. If a friend or family member were about to try this app, what would you tell them to help them get ready or set expectations?
4. How well do you think the chatbot helped support your mental health or emotional wellbeing? Please give examples.
5. Tell us your thoughts on the best way to use the chatbot. For example, as part of a daily routine, or more of a “when needed” tool.
6. In your own words, how does the chatbot compare to a human therapist? What are the pros, cons, or key differences you noticed?
7. Did you feel like you could trust the chatbot? Why or why not?
8. Did you feel emotionally connected or supported while using it?
9. If you could improve one thing about the chatbot, what would it be and why?
10. Is there anything else you want to share about your experience that we haven’t asked? Any big takeaways or lingering thoughts?

**Supplemental Figures**


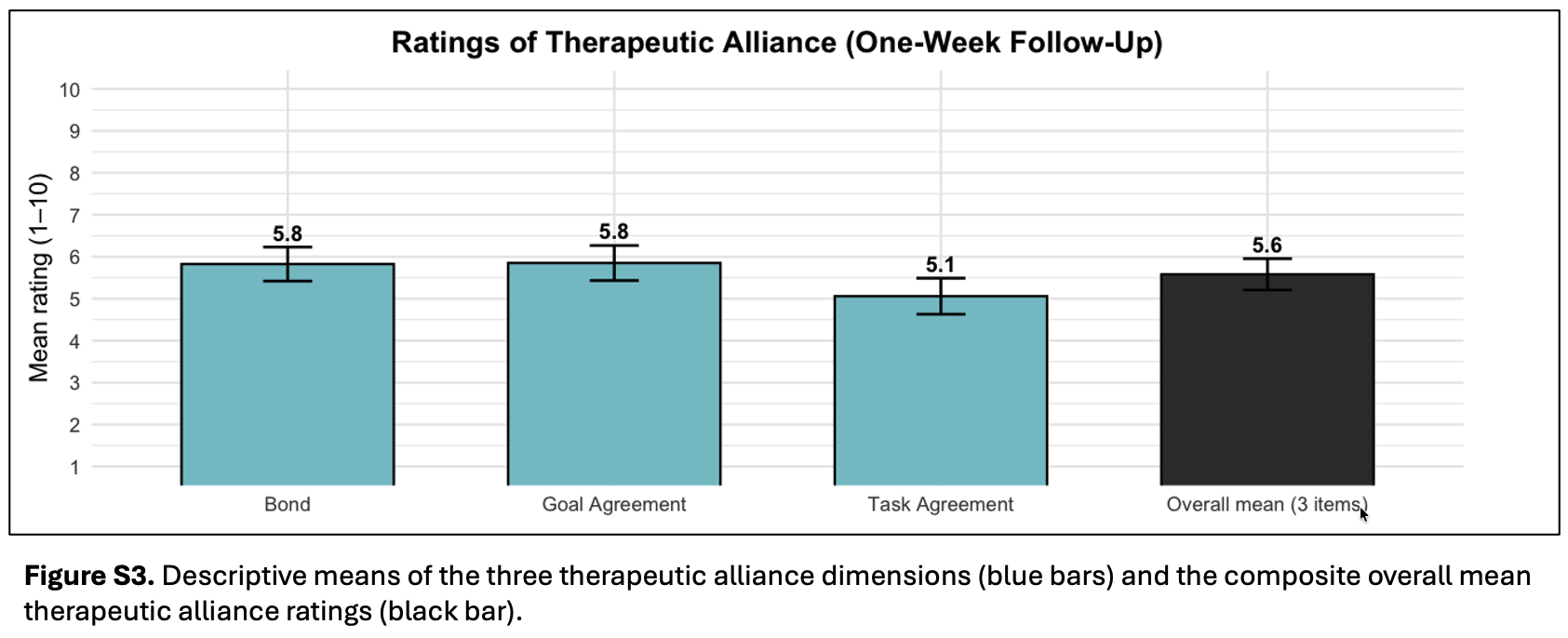


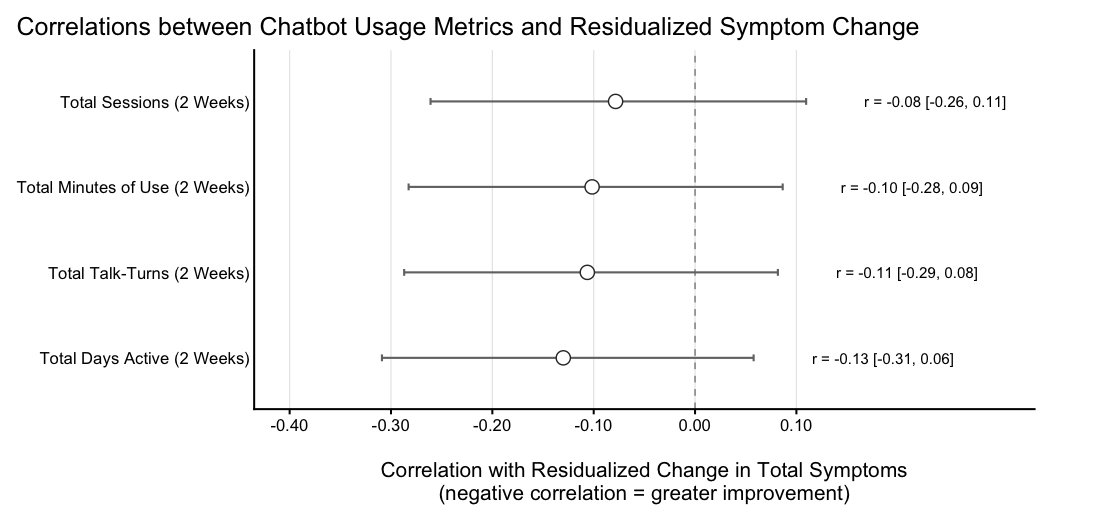


**Figure S4.** Correlations between user engagement metrics and symptom improvement (residualized change from baseline to 2-week follow-up).


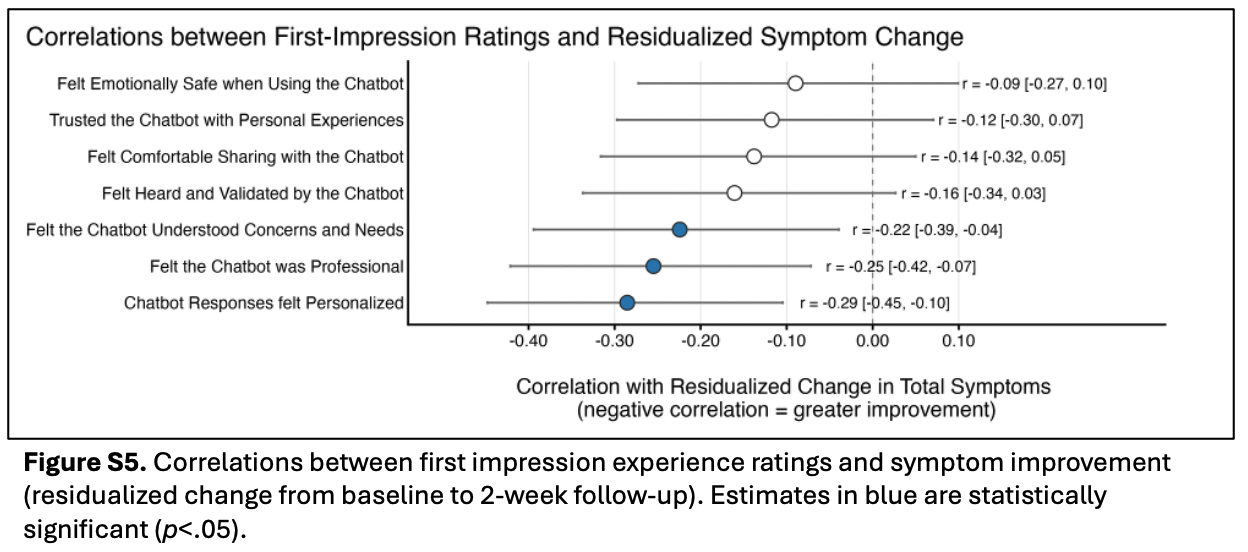


**Figure S5.** Correlations between first impression experience ratings and symptom improvement (residualized change from baseline to 2-week follow-up). Estimates in blue are statistically significant (*p* < .05).


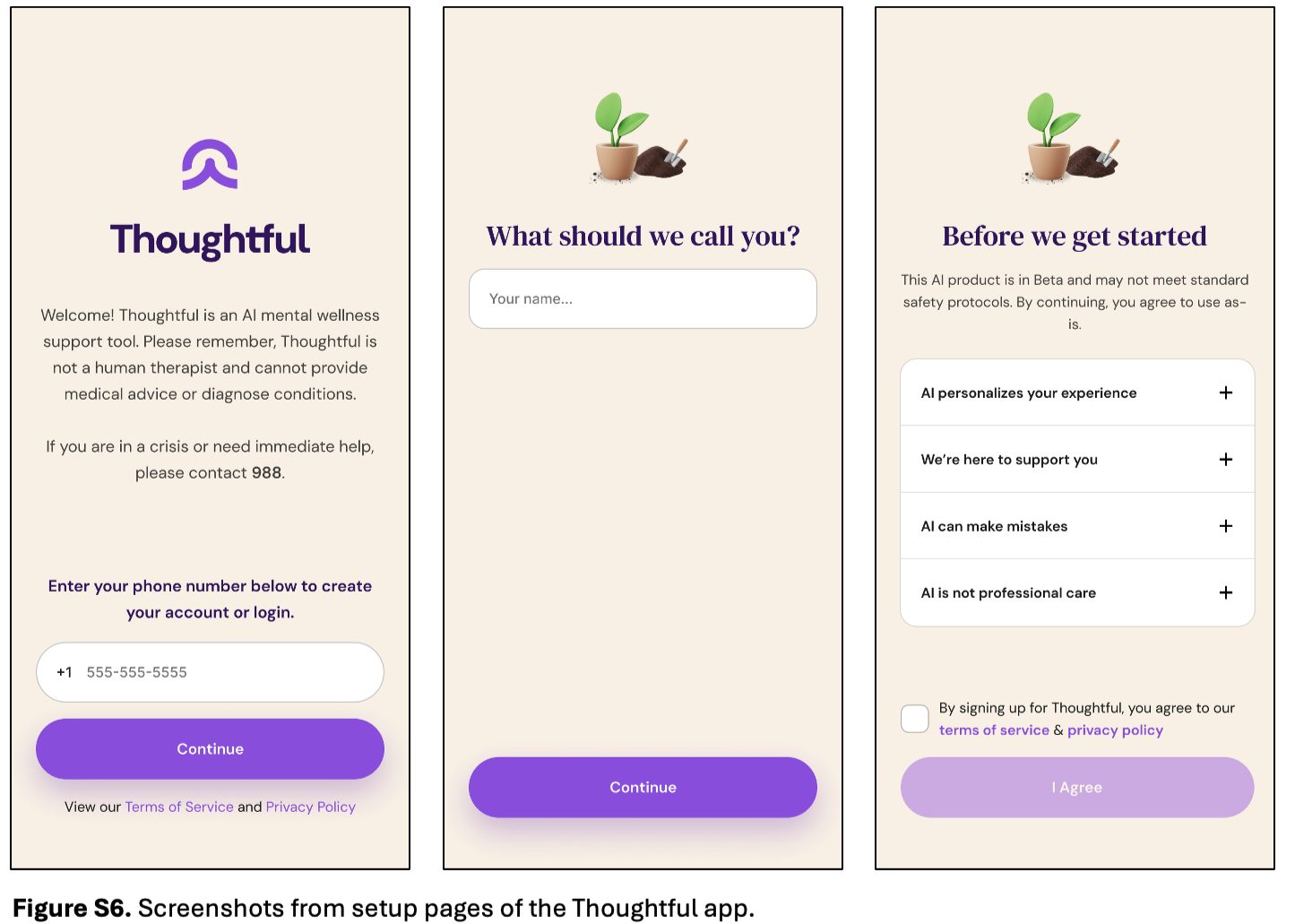


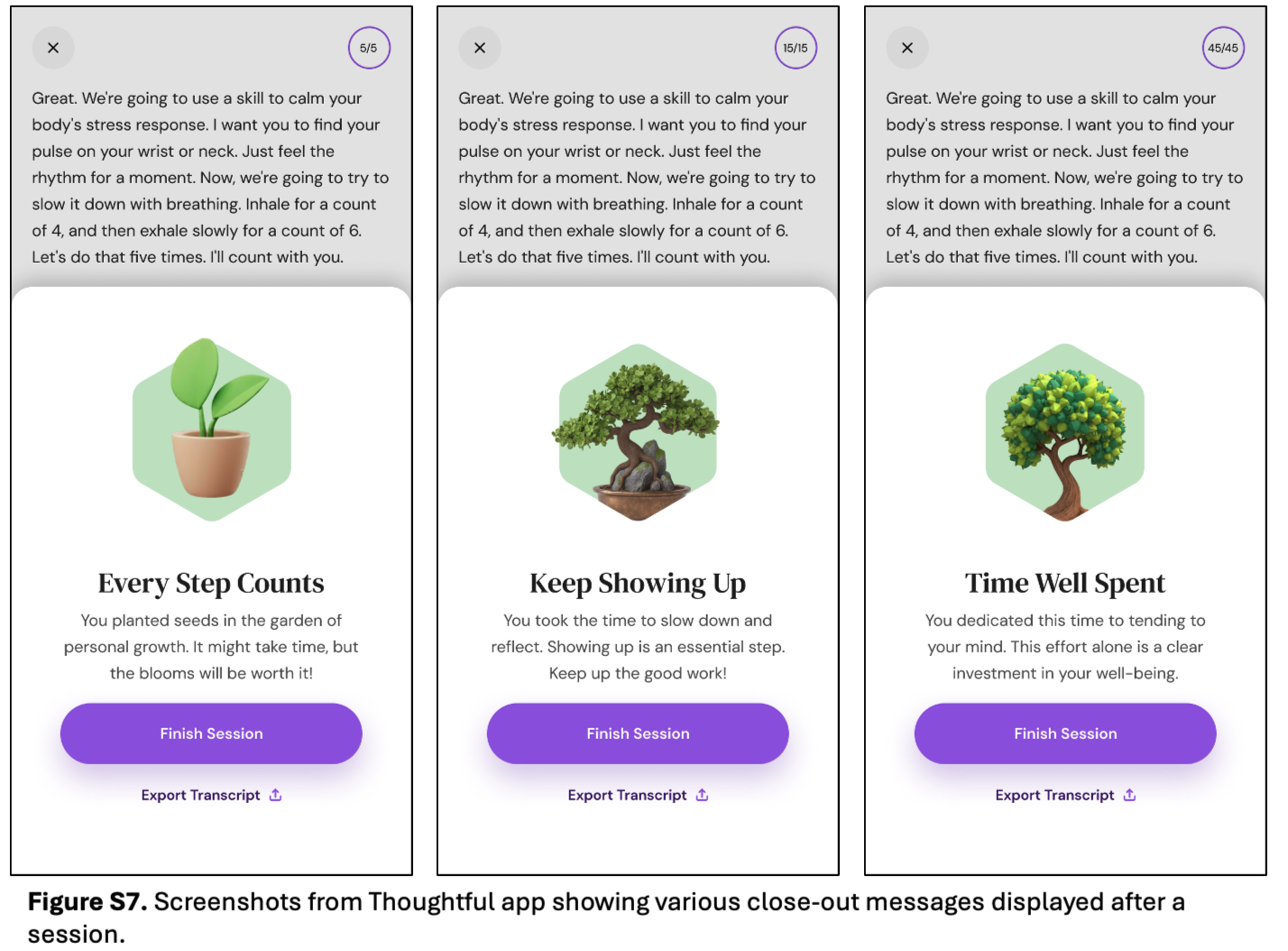


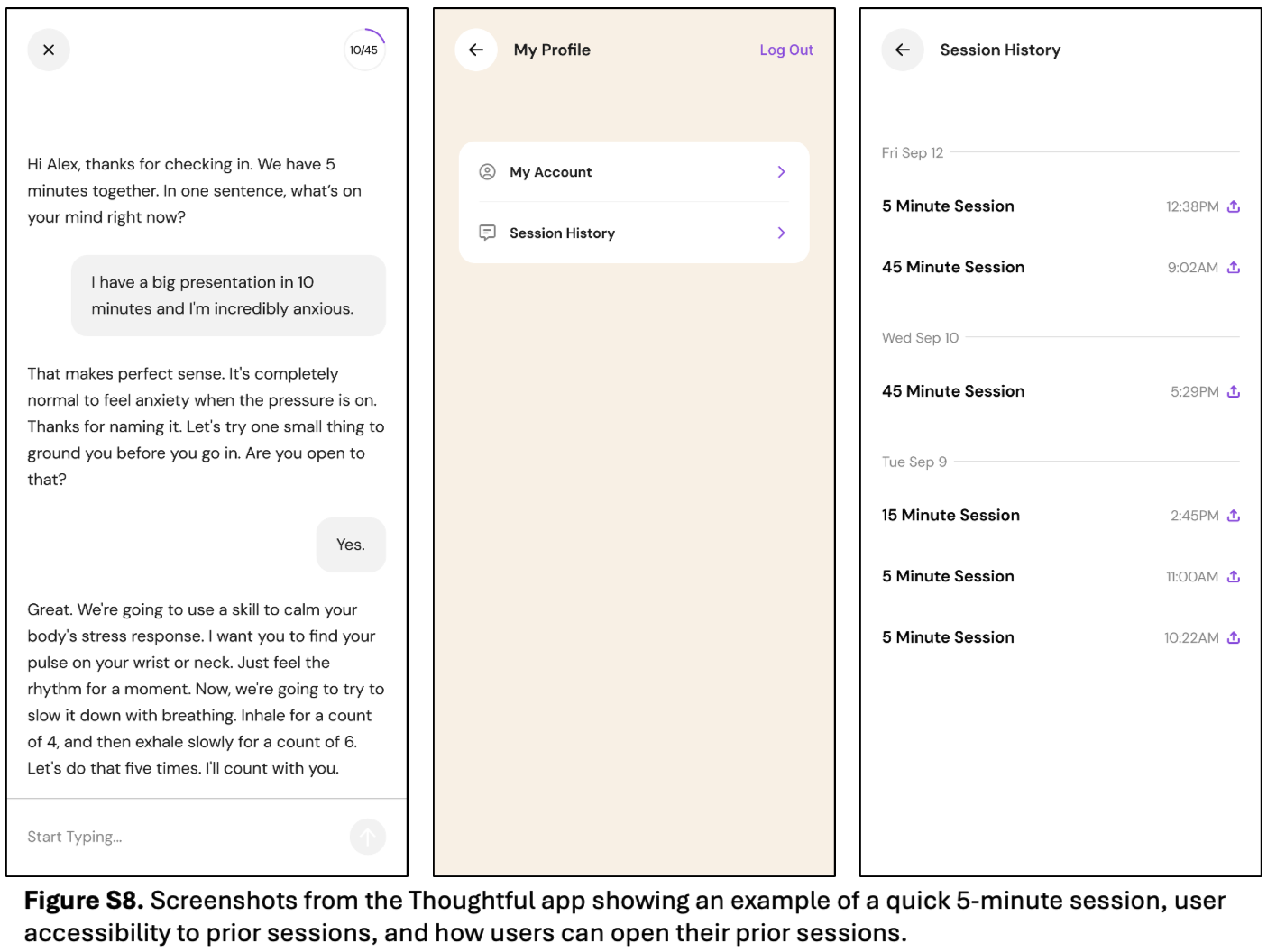


**References Cited in the Supplement**

1. Posner K, Brown GK, Stanley B, Brent DA, Yershova KV, Oquendo MA, Currier GW, Melvin GA, Greenhill L, Shen S, Mann JJ. The Columbia–Suicide Severity Rating Scale: Initial Validity and Internal Consistency Findings From Three Multisite Studies With Adolescents and Adults. Am J Psychiatry 2011 Dec;168(12):1266–1277. PMID:22193671

2. Kroenke K, Spitzer RL, Williams JBW, Löwe B. An ultra-brief screening scale for anxiety and depression: The PHQ-4. Psychosomatics Elsevier; 2009;50:613–621. doi: 10.1016/S0033-3182(09)70864-3

3. Kroenke K, Spitzer RL, Williams JBW. The PHQ-9: Validity of a brief depression severity scale. J Gen Intern Med 2001;16:606–613.

4. Wu Y, Levis B, Riehm KE, Saadat N, Levis AW, Azar M, Rice DB, Boruff J, Cuijpers P, Gilbody S, Ioannidis JPA, Kloda LA, McMillan D, Patten SB, Shrier I, Ziegelstein RC, Akena DH, Arroll B, Ayalon L, Baradaran HR, Baron M, Bombardier CH, Butterworth P, Carter G, Chagas MH, Chan JCN, Cholera R, Conwell Y, de Man-van Ginkel JM, Fann JR, Fischer FH, Fung D, Gelaye B, Goodyear-Smith F, Greeno CG, Hall BJ, Harrison PA, Härter M, Hegerl U, Hides L, Hobfoll SE, Hudson M, Hyphantis T, Inagaki M, Jetté N, Khamseh ME, Kiely KM, Kwan Y, Lamers F, Liu S-I, Lotrakul M, Loureiro SR, Löwe B, McGuire A, Mohd-Sidik S, Munhoz TN, Muramatsu K, Osório FL, Patel V, Pence BW, Persoons P, Picardi A, Reuter K, Rooney AG, Santos IS, Shaaban J, Sidebottom A, Simning A, Stafford L, Sung S, Lynnette Tan PL, Turner A, van Weert HC, White J, Whooley MA, Winkley K, Yamada M, Benedetti A, Thombs BD. Equivalency of the diagnostic accuracy of the PHQ-8 and PHQ-9: A systematic review and individual participant data meta-analysis. Psychol Med 2020 June;50(8):1368–1380. PMID:31298180

5. Spitzer RL, Kroenke K, Williams JBW, Löwe B. A brief measure for assessing generalized anxiety disorder: The GAD-7. Arch Intern Med 2006;166:1092–1097. PMID:16717171

6. Kroenke K, Baye F, Lourens SG. Comparative validity and responsiveness of PHQ-ADS and other composite anxiety-depression measures. J Affect Disord 2019 Mar 1;246:437–443. doi: 10.1016/j.jad.2018.12.098

7. Bech P, Gudex C, Johansen KS. The WHO (Ten) Weil-Being Index: Validation in Diabetes. Psychother Psychosom S. Karger AG; 1996;65(4):183–190.

8. Kessler RC, Andrews G, Colpe LJ, Hiripi E, Mroczek DK, Normand SLT, Walters EE, Zaslavksy AM. Short screening scales to monitor population prevalences and trends in non-specific psychological distress. Psychol Med 2002;32:959–976.

9. Hughes ME, Waite LJ, Hawkley LC, Cacioppo JT. A short scale for measuring loneliness in large surveys: Results from two population-based studies. Res Aging 2004;26:655–672. PMID:18504506

10. Schröder J, Sautier L, Kriston L, Berger T, Meyer B, Späth C, Köther U, Nestoriuc Y, Klein JP, Moritz S. Development of a questionnaire measuring Attitudes towards Psychological Online Interventions–the APOI. J Affect Disord 2015 Nov;187:136–141. doi: 10.1016/j.jad.2015.08.044

11. Sekhon M, Cartwright M, Francis JJ. Acceptability of healthcare interventions: an overview of reviews and development of a theoretical framework. BMC Health Serv Res 2017 Jan 26;17(1):88. doi: 10.1186/s12913-017-2031-8

12. Lyon AR, Pullmann MD, Jacobson J, Osterhage K, Al Achkar M, Renn BN, Munson SA, Areán PA. Assessing the usability of complex psychosocial interventions: The Intervention Usability Scale. Implement Res Pract SAGE Publications; 2021 Jan 1;2:2633489520987828. doi: 10.1177/2633489520987828
